# The global, regional, and national burden of gastro-oesophageal reflux disease in 195 countries and territories, 1990–2017: a systematic analysis for the Global Burden of Disease Study 2017

**DOI:** 10.1016/S2468-1253(19)30408-X

**Published:** 2020-03-13

**Authors:** GBD 2017 Gastro-oesophageal Reflux Disease Collaborators, M Ashworth Dirac, Saeid Safiri, Derrick Tsoi, Rufus Adesoji Adedoyin, Ashkan Afshin, Narjes Akhlaghi, Fares Alahdab, Abdulaziz M Almulhim, Saeed Amini, Floriane Ausloos, Umar Bacha, Maciej Banach, Akshaya Srikanth Bhagavathula, Ali Bijani, Antonio Biondi, Antonio Maria Borzì, Danny Colombara, Kathleen Elizabeth Corey, Baye Dagnew, Ahmad Daryani, Dragos Virgil Davitoiu, Feleke Mekonnen Demeke, Gebre Teklemariam Demoz, Huyen Phuc Do, Arash Etemadi, Farshad Farzadfar, Florian Fischer, Abadi Kahsu Gebre, Hadush Gebremariam, Berhe Gebremichael, Ahmad Ghashghaee, Uday C Ghoshal, Samer Hamidi, Milad Hasankhani, Shoaib Hassan, Simon I Hay, Chi Linh Hoang, Michael K Hole, Kevin S Ikuta, Olayinka Stephen Ilesanmi, Seyed Sina Naghibi Irvani, Spencer L James, Farahnaz Joukar, Ali Kabir, Hagazi Gebremedhin Kassaye, Taras Kavetskyy, Andre Pascal Kengne, Rovshan Khalilov, Muhammad U Khan, Ejaz Ahmad Khan, Maseer Khan, Amir Khater, Ruth W Kimokoti, Ai Koyanagi, Ana-Laura Manda, Dhruv Mehta, Varshil Mehta, Tuomo J Meretoja, Tomislav Mestrovic, Erkin M Mirrakhimov, Prasanna Mithra, Abdollah Mohammadian-Hafshejani, Milad Mohammadoo-Khorasani, Ali H Mokdad, Maryam Moossavi, Ghobad Moradi, Ghulam Mustafa, Mukhammad David Naimzada, Siavosh Nasseri-Moghaddam, Javad Nazari, Ionut Negoi, Cuong Tat Nguyen, Huong Lan Thi Nguyen, Molly R Nixon, Solomon Olum, Akram Pourshams, Hossein Poustchi, Mohammad Rabiee, Navid Rabiee, Alireza Rafiei, Salman Rawaf, David Laith Rawaf, Nicholas L S Roberts, Gholamreza Roshandel, Saeed Safari, Hamideh Salimzadeh, Benn Sartorius, Arash Sarveazad, Sadaf G Sepanlou, Amrollah Sharifi, Amin Soheili, Hafiz Ansar Rasul Suleria, Degena Bahrey Tadesse, Freweini Gebrearegay G Tela, Berhe Etsay Tesfay, Bhaskar Thakur, Bach Xuan Tran, Marco Vacante, Parviz Vahedi, Yousef Veisani, Theo Vos, Kia Vosoughi, Andrea Werdecker, Adam Belay Wondmieneh, Yordanos Gizachew Yeshitila, Mohammad Zamani, Kaleab Alemayehu Zewdie, Zhi-Jiang Zhang, Reza Malekzadeh, Mohsen Naghavi

## Abstract

**Background:**

Gastro-oesophageal reflux disease is a common chronic ailment that causes uncomfortable symptoms and increases the risk of oesophageal adenocarcinoma. We aimed to report the burden of gastro-oesophageal reflux disease in 195 countries and territories between 1990 and 2017, using data from the Global Burden of Diseases, Injuries, and Risk Factors Study (GBD) 2017.

**Methods:**

We did a systematic review to identify measurements of the prevalence of gastro-oesophageal reflux disease in geographically defined populations worldwide between 1990 and 2017. These estimates were analysed with DisMod-MR, a Bayesian mixed-effects meta-regression tool that incorporates predictive covariates and adjustments for differences in study design in a geographical cascade of models. Fitted values for broader geographical units inform prior distributions for finer geographical units. Prevalence was estimated for 195 countries and territories. Reports of the frequency and severity of symptoms among individuals with gastro-oesophageal reflux disease were used to estimate the prevalence of cases with no, mild to moderate, or severe to very severe symptoms at a given time; these estimates were multiplied by disability weights to estimate years lived with disability (YLD).

**Findings:**

Data to estimate gastro-oesophageal reflux disease burden were scant, totalling 144 location-years (unique measurements from a year and location, regardless of whether a study reported them alongside measurements for other locations or years) of prevalence data. These came from six (86%) of seven GBD super-regions, 11 (52%) of 21 GBD regions, and 39 (20%) of 195 countries and territories. Mean estimates of age-standardised prevalence for all locations in 2017 ranged from 4408 cases per 100 000 population to 14 035 cases per 100 000 population. Age-standardised prevalence was highest (>11 000 cases per 100 000 population) in the USA, Italy, Greece, New Zealand, and several countries in Latin America and the Caribbean, north Africa and the Middle East, and eastern Europe; it was lowest (<7000 cases per 100 000 population) in the high-income Asia Pacific, east Asia, Iceland, France, Denmark, and Switzerland. Global prevalence peaked at ages 75–79 years, at 18 820 (95% uncertainty interval [95% UI] 13 770–24 000) cases per 100 000 population. Global age-standardised prevalence was stable between 1990 and 2017 (8791 [95% UI 7772–9834] cases per 100 000 population in 1990 and 8819 [7781–9863] cases per 100 000 population in 2017, percentage change 0·3% [–0·3 to 0·9]), but all-age prevalence increased by 18·1% (15·6–20·4) between 1990 and 2017, from 7859 (6905–8851) cases per 100  000 population in 1990 to 9283 (8189–10 400) cases per 100  000 population in 2017. YLDs increased by 67·1% (95% UI 63·5–70·3) between 1990 and 2017, from 3·60 million (1·93–6·12) in 1990 to 6·01 million (3·22–10·19) in 2017.

**Interpretation:**

Gastro-oesophageal reflux disease is common worldwide, although less so in much of eastern Asia. The stability of our global age-standardised prevalence estimates over time suggests that the epidemiology of the disease has not changed, but the estimates of all-age prevalence and YLDs, which increased between 1990 and 2017, suggest that the burden of gastro-oesophageal reflux disease is nonetheless increasing as a result of ageing and population growth.

**Funding:**

Bill & Melinda Gates Foundation.

## Introduction

Gastro-oesophageal reflux disease is a common and usually chronic ailment of the upper digestive tract. Some reflux of stomach contents into the oesophagus, with or without symptoms, is physiological. Gastro-oesophageal reflux disease, however, is defined as a condition that develops when the reflux of stomach contents causes troublesome symptoms, complications, or both.^1^ Why some individuals have more frequent or severe symptoms or complications of reflux than others is poorly understood, but obesity, hiatal hernias, alcohol, smoking, and various foods and medications have been reported as risk factors.^2^, ^3^, ^4^ A positive association with age has been observed in many^4^—but not all^5^—studies.

**Research in context**

**Evidence before this study**

The Global Burden of Diseases, Injuries, and Risk Factors Study (GBD) has not estimated the burden of health loss due to gastro-oesophageal reflux disease. Two previous systematic reviews and one previous meta-analysis evaluated the prevalence of gastro-oesophageal reflux disease and its geographical variation. These studies suggested that the prevalence of this disease around the world ranged from 2·5% to 33·1%, and that prevalence was lower in east Asia and southeast Asia. One systematic review suggested that prevalence increased after 1995. The designs of these studies did not quantitatively account for the effect that differences in study design might have on study results, and only provided estimates of prevalence for the small number of countries where original studies have been done or for broadly defined regions, and did not estimate the burden of gastro-oesophageal reflux disease in terms of years lived with disability (YLDs) or other composite measures of health loss.

**Added value of this study**

GBD 2017 provides the first comprehensive estimates of global, regional, and country-specific prevalence and non-fatal health loss due to gastro-oesophageal reflux disease for 195 countries and territories, from 1990 to 2017, using patterns observed in data from different locations, ages, and times to produce the best possible estimates both where data are available and where they are not. GBD 2017 incorporated more data sources on the prevalence of gastro-oesophageal reflux disease than previous systematic reviews and meta-analyses, and used a modelling approach that adjusted for the effects of non-standard study designs on prevalence data. Even after these adjustments, GBD 2017 generally confirmed the findings reported in previous studies with regard to the range of gastro-oesophageal reflux disease prevalence seen worldwide and the finding that prevalence is lower in countries in east Asia and in the high-income Asia Pacific, but it did not find a global increase in the prevalence of gastro-oesophageal reflux disease after accounting for population ageing.

**Implications of all the available evidence**

Gastro-oesophageal reflux disease is common and increasing due to population ageing. Health-care systems should be prepared to address the needs of increasing numbers of patients with gastro-oesophageal reflux disease. In some locations, there might be an increase in the prevalence of gastro-oesophageal reflux disease beyond the increase due to age, but more research is required to determine whether this is true and, if so, what factors are driving this increase and what interventions might decrease the burden of gastro-oesophageal reflux disease.

Gastro-oesophageal reflux disease syndromes include typical reflux (defined by heartburn, regurgitation, or both, and sometimes accompanied by belching, water brash, or nausea), angina-mimicking chest pain, and extra-oesophageal symptoms such as chronic cough and chronic laryngitis.^1^, ^6^ Complications of gastro-oesophageal reflux disease include oesophageal inflammation, stricture,^7^ ulceration, perforation, metaplasia (ie, Barrett's oesophagus), and oesophageal adenocarcinoma.^8^, ^9^, ^10^, ^11^, ^12^ Associations of varying strength have been detected between reflux beyond the oesophagus and outcomes such as dental erosion,^13^ difficulty controlling concurrent asthma,^6^, ^14^ and increased risk of laryngopharyngeal carcinoma.^15^

Lifestyle changes to reduce reflux of stomach contents, such as weight loss and eating smaller meals, are commonly recommended (eg, by treating physicians and in practice guidelines written by professional organisations and committees) and moderately supported by evidence.^16^, ^17^ Often, however, effective control of symptoms requires the use of acid-suppressing medications, such as proton-pump inhibitors. Long-term use of proton-pump inhibitors has been associated with adverse outcomes such as loss of bone-mineral density and increased occurrence of enteric and pulmonary infections.^18^, ^19^, ^20^, ^21^, ^22^, ^23^, ^24^, ^25^, ^26^, ^27^ Surgical or endoscopic procedures to reduce reflux are done in selected medication-dependent or refractory cases. Health-care systems and individuals incur economic costs for physician visits, medications, and procedures.^28^, ^29^, ^30^, ^31^, ^32^, ^33^, ^34^, ^35^

Objective measures such as oesophageal pH monitoring or endoscopy can be used to diagnose gastro-oesophageal reflux disease or its effect on oesophageal mucosa, but these procedures are invasive and can miss cases with fluctuating course. Multiple expert groups have endorsed the use of clinical history and response to therapy in making a clinical diagnosis.^1^, ^36^ Multiple symptom-based questionnaires have been developed for use in population-based research,^37^ and prevalence studies have mainly been carried out with this approach.

Several systematic reviews have been published in the past two decades describing the incidence and prevalence of gastro-oesophageal reflux disease.^38^, ^39^ The methodology of systematic reviews, however, limits comparisons across geography and time to those geographies and times for which reported studies exist, and does not quantitatively account for differences in study design. Eusebi and colleagues did a meta-analysis of gastro-oesophageal reflux disease,^4^ which produced global and regional pooled estimates of disease prevalence and explored features of study designs that might explain inter-study heterogeneity, but did not use information about these design features to adjust the contribution of non-standard studies to pooled estimates. Furthermore, the chronicity of gastro-oesophageal reflux disease and the fact that it can cause persistent or episodic symptoms of varying severity make it important to move beyond estimations of incidence and prevalence, and to quantify the severity and duration of health loss it causes. The Global Burden of Disease research framework uses meta-regression methods to synthesise data from published studies to make estimates for 195 countries and territories worldwide from 1990 to the present, and expresses the relative health loss due to more than 350 diseases and injuries in common terms that facilitate comparisons. Here, we report results from the Global Burden of Diseases, Injuries, and Risk Factors Study (GBD) 2017, the first iteration of GBD to estimate non-fatal health loss due to gastro-oesophageal reflux disease.

## Methods

### Overview

The overall objectives, methods, and organisation of GBD 2017 have been previously reported.^40^, ^41^, ^42^ Methods relevant to estimating the burden of gastro-oesophageal reflux disease are summarised here and described further in the appendix (pp 1–8).

For our analysis, individuals with heartburn, regurgitation, or both, at least once weekly over a 12-month recall period, were defined as having gastro-oesophageal reflux disease. This definition was chosen over the consensus-group-recommended definition of mild symptoms occurring at least twice a week or moderate to severe symptoms occurring at least weekly^1^ because of greater data availability, and is consistent with a previously published meta-analysis.^4^ Individuals who had oesophageal complications (eg, ulceration or metaplasia) without symptoms, whose sole symptom of gastro-oesophageal reflux was chest pain without typical reflux symptoms, or who had reflux primarily as a trigger or exacerbating factor in respiratory or head and neck diseases (eg, chronic cough or dental erosion) were not included. This strategy avoids double-counting disability already attributed to other underlying diseases modelled in GBD.

### Prevalence estimation

Data inputs for estimating the prevalence of gastro-oesophageal reflux disease included epidemiological studies of gastrointestinal illness published in peer-reviewed journals and identified in a systematic review via PubMed, and data from the US National Health Interview Surveys. Search terms and other details of the systematic review are provided in the appendix (pp 1–3). A complete set of unadjusted input data included in the model can be downloaded from the GBD 2017 Data Resources website. Extracted data from studies with acceptable but non-preferred designs were marked with study-level covariates to allow for estimation of fixed effects due to study characteristics in our global meta-regression analysis (described later).

Gastro-oesophageal reflux disease data were analysed with a Bayesian mixed-effects meta-regression framework, DisMod-MR 2.1, developed for GBD non-fatal estimation processes, which has been previously described in detail^42^, ^43^, ^44^ and is summarised here. Estimates are made by fitting a series of models, each of which serves to generate a Bayesian prior distribution for a subsequent model. At each step, DisMod assumes a compartmental disease model with three states—susceptible, diseased, and dead—with transition between states determined by incidence, remission, excess mortality due to disease, and other-cause mortality. These disease parameters are modelled with an offset log-normal data likelihood function, and a system of age-integrated differential equations are solved to ensure internal consistency among disease parameters.

The first model in the DisMod series is a global mixed-effect model, which uses all data from both sexes, all locations, and all years, and estimates coefficients for fixed effects for sex, study design characteristics, and predictive covariates, and random effects for each of the seven GBD super-regions. The next step is to fit separate mixed-effects models for each year, sex, and super-region, each of which re-estimates the fixed effect coefficients and estimates random effects for each GBD region within that super-region; the Bayesian prior distribution for each super-region-level model is based on the distribution estimated by the initial global model with the fixed effects and the random effect for that super-region. This method is repeated to fit separate mixed-effects models specific to sex, year, and region, using the preceding super-region model and the random effect for the region to determine the Bayesian prior, and estimating random effects for countries. This approach is again repeated to fit separate models specific to sex, year, and country, using the preceding regional model and the random effect for the country to determine the Bayesian prior. For 15 countries, an additional round of models is fit for subnational units (such as states or provinces), each deriving its Bayesian prior from its country model and a pseudo-random effect based on the average ratio of observed subnational data to country-model predictions. This algorithm for developing prior distributions for subnational models is sensitive to data in age groups that have low estimated values in the country-level fit, which can cause the model to ignore the preponderance of the data; in these cases, data for the affected age groups in the subnational locations are excluded.

As mentioned, the DisMod framework estimates fixed effects for study design characteristics; these study-level fixed effects reflect the association observed in input data between study design characteristics and measured disease parameters, and they serve to adjust for measurement bias due to non-reference study designs. Fixed effects are also estimated for predictive covariates; these reflect the association observed between that covariate and disease input data and serve to help estimate disease parameters in locations with scarce or absent input data. To be considered as a predictive covariate, a factor must have a demonstrated association with disease in non-GBD studies, and valid estimates of the distribution of that factor must exist for all GBD locations and estimation years available to use as DisMod inputs.^42^ The association between a predictive covariate and disease parameters need not be causal to serve this purpose. Candidate predictive covariates found to have null or highly uncertain coefficients in preliminary models do not improve estimates, so they are left out of the final model for parsimony.

Ultimately, final estimates for national or subnational locations reflect local data, adjusted for study design characteristics, if local data are present, and reflect prior distributions from broader geographical units and the influence of predictive covariates if no local data are available. Estimates from the finest level of geography are later aggregated to make final estimates for the broader geographical units. Uncertainty intervals are taken as the 2·5th and 97·5th percentiles of the posterior distribution.

Parameters used in DisMod for gastro-oesophageal reflux disease were as follows: excess mortality was assumed a priori to be 0, and remission prior was set to 0·2–0·5 cases per person-year. Incidence was forced to 0 from birth to age 5 years, and after this age prior was set to 0·0–0·2 cases per person-year. We included study-level covariates for alternative recall periods, for alternative minimum symptom frequencies, for the use of a score-based case definition that synthesised the severity, number, and frequency of symptoms, for the use of a case definition based on a single cardinal reflux symptom (regurgitation only), for studies in which the representativeness of the sample was considered questionable, and for data extracted from a report from a national survey, rather than a peer-reviewed publication. We considered location-level covariates for mean body-mass index (BMI), smoking prevalence, mean alcohol consumption,^45^ and the Healthcare Access and Quality Index,^46^ but these covariates were non-predictive in preliminary models, so they were not retained in the final model.

### Estimation of years lived with disability

Years lived with disability (YLDs) synthesise the frequency and non-fatal health consequences of a disease. YLD estimation in GBD^42^ begins by estimating the point prevalence, specific to year, age, sex, and location, of specific health states that can result from the disease, generally at different levels of severity. Each of these disease states corresponds to one of a set of health states for which disability weights have been derived from population-based surveys.^47^, ^48^, ^49^ Health states describe the consequences of disease or injury in terms relevant to an individual's life, such as loss of function and pain or other symptoms. The disability weights for these health states range from 0 to 1, with 0 representing perfect health and 1 representing death. Prevalent cases in each health state are multiplied by the disability weight of that health state to calculate YLDs. In a microsimulation process, all health states for all diseases are assigned to simulants according to their point-prevalence specific to year, age, sex, and location, assuming independent probability. For simulants assigned health states for multiple diseases, YLDs are adjusted with a multiplicative function of the disability weights. YLDs due to all health states of each disease are then summed.

The prevalence of health states for gastro-oesophageal reflux disease was determined from severity and frequency distributions reported in the prevalence studies used in our prevalence model. Severity and frequency categories were combined, as described below, to generate four categories, and these categories were assigned the health states and disability weights shown in the appendix (p 5).

Throughout the literature, the severity of gastro-oesophageal reflux disease is often divided into two to five categories according to diverse definitions. We reviewed the studies in our input data and, if provided, extracted counts of cases of each severity as reported. These cases were then mapped to one of two GBD 2017 gastro-oesophageal reflux disease severities: mild to moderate (disability weight 0·011; 95% uncertainty interval [95% UI] 0·005–0·021) and severe to very severe (disability weight 0·027; 0·015–0·046).^47^, ^48^, ^49^ The proportion of cases in each of the GBD 2017 gastro-oesophageal reflux disease severities was calculated for the pooled total cases, along with standard errors based on a simple proportion model.

Many studies also report the frequency of gastro-oesophageal reflux disease symptoms as the proportions of cases in each of a set of mutually exclusive and collectively exhaustive frequency categories. Examples include 1–6 days per week and daily; 1 day per week, 2–6 days per week, and daily; 1–3 days per week, 4–6 days per week, and daily; and so on. For each study, 1000 proportion draws were generated for each frequency category with a beta distribution. These proportion draws were multiplied by the assumed mean days per week symptomatic for the category (the midpoint of the range) to produce draws of the number of days per week symptomatic that were contributed by cases in that category, and these draws for proportion-weighted means were summed across categories to estimate days per week symptomatic for all cases in the study. Means and SDs of these draws were combined in a meta-analysis, and the final mean and SD were divided by seven to estimate the proportion of cases that were symptomatic on a given day, with uncertainty.

Data about severity and frequency were too sparse to adjust meta-analyses for person, place, or time, so the same pooled proportions were applied to all combinations of year, age, sex, and location.

Because a single distribution of severity and frequency was applied to calculate YLDs for all years, ages, sexes, and locations, all variation in YLDs is driven by variation in prevalence. Because fatalities related to gastro-oesophageal reflux disease are attributed to other underlying causes of death (eg, oesophageal carcinoma), no years of life lost (YLLs) are directly estimated for gastro-oesophageal reflux disease and disability-adjusted life-years (DALYs) are equal to YLDs.

Final estimates of prevalence and YLDs were specific to year, age, sex, and location. These estimates were weighted and aggregated by the age-sex distribution of the population in the location and year to which the estimates applied to produce all-age estimates. The same year-age-sex-location-specific estimates were adjusted to the GBD reference population by direct methods as previously described to produce age-standardised estimates.^50^, ^51^, ^52^

The percentage change in estimates between 1990 and 2017 was estimated by calculating the percentage change between pairs of 1000 draws from the bootstrap distributions of estimates for each year, then finding the mean and 25th and 975th ordered values of the resulting combined distribution.

At the recommendation of GBD network collaborators, as a post-hoc analysis, final age-standardised YLD rate estimates were plotted against GBD estimates of Socio-demographic Index^42^ and their relationships modelled with reduced cubic splines.

We documented each step of the GBD 2017 estimation processes, as well as data sources, in accordance with the Guidelines for Accurate and Transparent Health Estimates Reporting (GATHER) statement.

### Role of the funding source

The funder of the study had no role in study design, data collection, data analysis, data interpretation, or writing of the report. The corresponding authors had full access to all the data in the study and had final responsibility for the decision to submit for publication.

## Results

**Figure 1:**
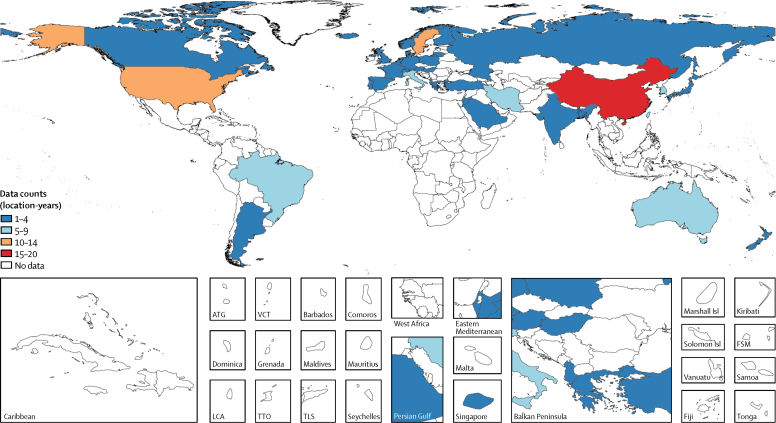
Prevalence data inputs for the non-fatal model of gastro-oesophageal reflux disease, 1990–2017 ATG=Antigua and Barbuda. VCT=Saint Vincent and the Grenadines. LCA=Saint Lucia. TTO=Trinidad and Tobago. TLS=Timor-Leste. Isl=Islands. FSM=Federated States of Micronesia.

In our systematic review, we found 112 studies that met the inclusion criteria. Four studies used diagnostic codes to identify cases in administrative data, two studies used self-reported diagnosis, and the remainder were surveys that used symptom-based questionnaires: 27 studies used the GBD case-definition for gastro-oesophageal reflux disease, and 79 studies used one of more than 50 alternatives that differed in recall period, minimum symptom frequency, defining symptoms, or manner of scoring. Combined with data from a household survey, this strategy provided 144 location-years of prevalence data and 406 prevalence datapoints; six datapoints in young age groups in subnational locations were excluded to avoid over-estimation of pseudorandom effects, as described above. Data for the model were from six (86%) of seven GBD super-regions, 11 (52%) of 21 GBD regions, and 39 (20%) of 195 countries and territories (figure 1). No data were found for southeast Asia, Oceania, central Asia, the Caribbean, Andean Latin America, central Latin America, or any region of sub-Saharan Africa. Data counts such as these for all diseases are found in the disease-specific summaries in the methods appendix of the GBD 2017 paper on non-fatal disease burden estimation.^42^

The estimates of age-standardised prevalence of gastro-oesophageal reflux disease for all countries and territories in GBD 2017 are presented in the table.

**Table:** Prevalence of gastro-oesophageal reflux disease in 1990 and 2017 for both sexes and all locations, with percentage change

		**1990**		**2017**		**Percentage change in age-standardised prevalence between 1990 and 2017**
		Cases (95% UI)	Age-standardised prevalence per 100 000 population (95% UI)	Cases (95% UI)	Age-standardised prevalence per 100 000 population (95% UI)	
**Global**		**423 963 525 (372 479 898 to 477 479 631)**	**8790·6 (7771·5 to 9834·2)**	**709 264 333 (625 708 694 to 794 604 211)**	**8818·9 (7780·9 to 9863·1)**	**0·3% (−0·3 to 0·9)**
**Central Europe, eastern Europe, and central Asia**		**52 103 922 (46 226 738 to 58 472 716)**	**11 411·5 (10 112·4 to 12 781·0)**	**58 613 496 (52 059 744 to 65 685 580)**	**11 358·2 (10 067·3 to 12 722·1)**	**−0·5% (−0·7 to −0·2)**
Central Asia		6 364 907 (5 583 352 to 7 177 563)	10 877·4 (9603·3 to 12 219·3)	9 801 342 (8 578 573 to 11 065 856)	10 880·0 (9602·8 to 12 224·1)	0% (−0·1 to 0·1)
	Armenia	352 696 (309 406 to 398 280)	10 878·0 (9601·4 to 12 216·0)	397 013 (350 172 to 446 718)	10 878·3 (9603·4 to 12 217·8)	0% (−0·1 to 0·1)
	Azerbaijan	697 585 (612 891 to 788 063)	10 876·1 (9604·6 to 12 215·2)	1 202 222 (1 054 666 to 1 359 421)	10 880·6 (9603·6 to 12 226·7)	0% (−0·1 to 0·2)
	Georgia	648 743 (573 578 to 728 695)	10 875·3 (9605·9 to 12 214·1)	499 792 (441 796 to 559 981)	10 879·3 (9603·6 to 12 221·3)	0% (−0·1 to 0·1)
	Kazakhstan	1 689 153 (1 482 763 to 1 906 743)	10 875·7 (9607·9 to 12 212·8)	2 025 117 (1 781 135 to 2 283 550)	10 877·2 (9605·8 to 12 218·7)	0% (−0·1 to 0·1)
	Kyrgyzstan	396 532 (347 972 to 447 107)	10 877·3 (9604·1 to 12 221·7)	631 286 (551 764 to 713 458)	10 879·6 (9603·3 to 12 222·6)	0% (−0·1 to 0·1)
	Mongolia	167 885 (146 201 to 191 726)	10 882·7 (9595·6 to 12 232·7)	345 751 (300 606 to 394 567)	10 879·8 (9602·5 to 12 224·0)	0% (−0·1 to 0·1)
	Tajikistan	414 349 (361 725 to 470 714)	10 881·3 (9594·9 to 12 226·5)	844 122 (737 391 to 961 429)	10 884·3 (9595·9 to 12 241·7)	0% (−0·1 to 0·2)
	Turkmenistan	299 365 (260 810 to 340 823)	10 878·1 (9600·5 to 12 220·2)	527 679 (461 060 to 596 888)	10 884·7 (9601·9 to 12 240·1)	0·1% (−0·1 to 0·2)
	Uzbekistan	1 698 598 (1 480 911 to 1 931 215)	10 879·1 (9599·5 to 12 222·1)	3 328 360 (2 909 666 to 3 774 888)	10 880·5 (9602·0 to 12 227·0)	0% (−0·1 to 0·1)
Central Europe		13 008 328 (11 436 224 to 14 614 575)	9384·7 (8250·4 to 10 543·7)	14 435 221 (12 760 673 to 16 161 908)	9396·7 (8254·6 to 10 552·4)	0·1% (0 to 0·3)
	Albania	260 382 (225 794 to 296 366)	9268·5 (8101·9 to 10 430·3)	309 922 (274 352 to 347 498)	9168·8 (8056·8 to 10 279·7)	−1·1% (−5·2 to 2·7)
	Bosnia and Herzegovina	439 944 (385 200 to 500 245)	9288·3 (8145·3 to 10 486·1)	420 554 (370 832 to 474 439)	9290·1 (8147·3 to 10 491·3)	0% (−0·1 to 0·1)
	Bulgaria	999 730 (878 178 to 1 126 507)	9289·3 (8146·1 to 10 490·6)	915 565 (808 113 to 1 027 715)	9291·0 (8149·6 to 10 493·1)	0% (−0·1 to 0·1)
	Croatia	542 018 (477 567 to 613 473)	9285·4 (8141·6 to 10 487·0)	542 617 (480 253 to 610 300)	9289·7 (8147·2 to 10 490·9)	0% (−0·1 to 0·1)
	Czech Republic	1 111 875 (978 371 to 1 250 731)	9285·6 (8140·7 to 10 485·0)	1 334 726 (1 178 090 to 1 499 650)	9292·1 (8151·6 to 10 495·8)	0·1% (0 to 0·2)
	Hungary	1 133 724 (991 109 to 1 273 814)	9029·3 (7868·8 to 10 191·3)	1 200 586 (1 052 677 to 1 349 429)	9029·5 (7870·9 to 10 191·5)	0% (0 to 0·1)
	North Macedonia	191 727 (167 838 to 217 946)	9291·6 (8150·0 to 10 492·1)	259 906 (229 700 to 293 218)	9294·5 (8155·1 to 10 497·5)	0% (0 to 0·1)
	Montenegro	60 782 (53 290 to 68 863)	9288·4 (8143·7 to 10 484·4)	73 077 (64 443 to 82 302)	9290·0 (8147·9 to 10 489·6)	0% (0 to 0·1)
	Poland	4 121 732 (3 614 649 to 4 630 101)	9676·8 (8476·6 to 10 878·0)	4 958 070 (4 370 086 to 5 558 001)	9678·2 (8470·6 to 10 884·2)	0% (0 to 0·1)
	Romania	2 405 274 (2 118 210 to 2 718 809)	9288·1 (8144·4 to 10 488·7)	2 420 991 (2 129 819 to 2 720 958)	9290·3 (8148·5 to 10 492·3)	0% (0 to 0·1)
	Serbia	997 193 (878 033 to 1 129 595)	9290·3 (8146·7 to 10 490·2)	1 070 416 (945 234 to 1 202 930)	9291·0 (8149·2 to 10 492)	0% (0 to 0)
	Slovakia	529 557 (465 433 to 597 095)	9285·3 (8138·5 to 10 482·5)	664 826 (585 386 to 750 034)	9289·0 (8146·7 to 10 491·1)	0% (0 to 0·1)
	Slovenia	214 392 (188 240 to 242 437)	9285·1 (8142·1 to 10 486·7)	263 968 (233 260 to 296 728)	9294·6 (8155·2 to 10 498·1)	0·1% (−0·1 to 0·3)
Eastern Europe		32 730 687 (29 086 731 to 36 680 186)	12 622·0 (11 229·5 to 14 143·0)	34 376 933 (30 506 544 to 38 563 945)	12 618·8 (11 223·3 to 14 138·4)	0% (−0·1 to 0·1)
	Belarus	1 489 446 (1 321 676 to 1 684 997)	12 550·9 (11 092·7 to 14 183·1)	1 556 424 (1 375 573 to 1 757 384)	12 551·2 (11 097·6 to 14 182·6)	0% (−0·1 to 0·1)
	Estonia	227 569 (201 686 to 257 020)	12 551·0 (11 092·4 to 14 183·3)	219 641 (195 214 to 246 925)	12 552·1 (11 098·4 to 14 187·7)	0% (−0·1 to 0·1)
	Latvia	398 581 (353 098 to 449 828)	12 550·8 (11 090·5 to 14 184·4)	330 733 (293 953 to 372 637)	12 551·5 (11 097·4 to 14 183·5)	0% (−0·1 to 0·1)
	Lithuania	551 993 (491 349 to 620 910)	13 073·7 (11 620·1 to 14 710·4)	503 815 (448 579 to 563 736)	13 176·8 (11 672·9 to 14 827·1)	0·8% (−3·1 to 5·2)
	Moldova	577 391 (509 627 to 651 054)	12 550·0 (11 094·8 to 14 182·2)	598 746 (529 089 to 676 893)	12 551·5 (11 097·8 to 14 187·3)	0% (−0·1 to 0·1)
	Russia	21 439 973 (19 024 277 to 24 064 971)	12 493·1 (11 118·2 to 13 951·4)	23 453 080 (20 787 115 to 26 286 224)	12 501·5 (11 126·5 to 13 971·0)	0·1% (−0·1 to 0·2)
	Ukraine	8 045 735 (7 168 951 to 9 006 047)	12 975·7 (11 539·4 to 14 591·5)	7 714 495 (6 859 516 to 8 648 330)	12 976·0 (11 536·9 to 14 591·8)	0% (−0·1 to 0·1)
**High income**		**94 282 055 (83 699 292 to 105 034 547)**	**8889·8 (7868·5 to 9917·5)**	**130 784 112 (116 261 197 to 145 447 546)**	**9344·5 (8271 to 10 426·2)**	**5·1 % (3·3 to 6·9)**
Australasia		1 965 726 (1 739 701 to 2 194 756)	8733·1 (7729·1 to 9749·4)	3 078 914 (2 740 088 to 3 438 082)	8678·3 (7681·3 to 9690·9)	−0·6% (−0·8 to −0·5)
	Australia	1 491 398 (1 317 739 to 1 665 944)	7938·6 (6994·4 to 8927·5)	2 381 585 (2 105 621 to 2 661 501)	7943·2 (7001·3 to 8926·6)	0·1% (0 to 0·1)
	New Zealand	474 328 (419 642 to 534 071)	12 741·7 (11 255·1 to 14 393·9)	697 329 (618 925 to 778 044)	12 745·7 (11 269·5 to 14 394·5)	0% (−0·1 to 0·1)
High-income Asia Pacific		9 665 737 (8 443 271 to 10 850 283)	4859·3 (4260·0 to 5445·7)	13 910 586 (12 420 425 to 15 473 538)	5191·6 (4635·4 to 5793·9)	6·8% (4·2 to 10·5)
	Brunei	11 549 (9981 to 13 241)	5604·4 (4934·7 to 6319·3)	24 969 (21 680 to 28 406)	5577·8 (4912·0 to 6295·9)	−0·5% (−0·6 to −0·3)
	Japan	6 716 173 (5 883 553 to 7 547 327)	4377·1 (3824·9 to 4928·2)	8 450 935 (7 443 302 to 9 496 628)	4407·9 (3852·5 to 4957·5)	0·7% (0·6 to 0·8)
	Singapore	220 535 (192 203 to 251 088)	6697·7 (5878·4 to 7548·6)	472 967 (413 345 to 534 040)	6815·3 (5957·8 to 7685·1)	1·8% (−1·7 to 6·2)
	South Korea	2 717 480 (2 365 726 to 3 088 877)	6360·8 (5570·0 to 7155·7)	4 961 715 (4 446 577 to 5 473 438)	6841·5 (6140·3 to 7501·1)	7·6% (1·1 to 16·6)
High-income North America		37 381 801 (33 168 376 to 41 798 321)	11 708·9 (10 346·6 to 13 127·4)	55 883 926 (49 654 520 to 62 268 111)	12 346·1 (10 975·4 to 13 857·6)	5·4% (1·7 to 9·3)
	Canada	3 147 774 (2 755 775 to 3 524 033)	10 074·9 (8827·1 to 11 271·9)	4 703 994 (4 149 115 to 5 286 943)	10 076·2 (8829·7 to 11 274·4)	0% (0 to 0·1)
	Greenland	6094 (5310 to 6989)	10 906·5 (9621·3 to 12 286·9)	7154 (6293 to 8095)	10 889·0 (9606·9 to 12 280·0)	−0·2% (−0·4 to 0·1)
	USA	34 227 096 (30 352 163 to 38 318 050)	11 888·2 (10 502·9 to 13 327·0)	51 171 800 (45 550 645 to 57 025 039)	12 608·2 (11 205·8 to 14 166·5)	6·1% (1·9 to 10·3)
Southern Latin America		5 165 628 (4 525 027 to 5 810 197)	10 745·2 (9446·6 to 12 101·4)	7 895 164 (6 947 483 to 8 840 351)	10 742·8 (9445·5 to 12 097·9)	0% (0 to 0)
	Argentina	3 472 219 (3 046 169 to 3 904 100)	10 744·2 (9445·3 to 12 100·2)	5 198 657 (4 577 890 to 5 828 657)	10 743·4 (9445·9 to 12 099·3)	0% (0 to 0)
	Chile	1 329 485 (1 165 165 to 1 496 629)	10 747·5 (9449·2 to 12 103·9)	2 259 677 (1 982 387 to 2 539 740)	10 739·8 (9443·4 to 12 092·7)	−0·1% (−0·1 to 0)
	Uruguay	363 715 (321 422 to 406 761)	10 745·6 (9447·8 to 12 101·5)	436 480 (386 610 to 487 814)	10 748·8 (9450·7 to 12 106·9)	0% (0 to 0)
Western Europe		40 103 163 (35 710 201 to 44 581 113)	8490·7 (7516·1 to 9436·2)	50 015 523 (44 228 842 to 55 675 055)	8589·5 (7558·7 to 9567·2)	1·2% (−0·1 to 3·3)
	Andorra	5361 (4669 to 6055)	8398·9 (7333·0 to 9459·0)	9034 (7889 to 10 211)	8392·0 (7330·7 to 9455·3)	−0·1% (−0·2 to 0·1)
	Austria	946 297 (823 900 to 1 079 148)	9845·9 (8522·6 to 11 234·7)	1 174 844 (1 027 188 to 1 329 478)	9844·3 (8517·1 to 11 231·5)	0% (−0·2 to 0·1)
	Belgium	997 909 (885 765 to 1 112 948)	8039·8 (7075·2 to 8972·8)	1 221 408 (1 079 928 to 1 366 345)	8189·2 (7184·6 to 9198·3)	1·9% (−2·1 to 6·5)
	Cyprus	69 297 (60 616 to 78 051)	8394·7 (7333·4 to 9455·3)	135 309 (118 370 to 152 657)	8389·4 (7329·0 to 9451·1)	−0·1% (−0·2 to 0)
	Denmark	457 490 (405 015 to 511 617)	7127·3 (6276·6 to 8013·2)	517 335 (460 652 to 579 336)	6901·5 (6126·2 to 7740·0)	−3·2% (−9·2 to 1·2)
	Finland	506 254 (447 878 to 564 519)	8259·4 (7283·9 to 9239·1)	628 450 (558 783 to 703 706)	8429·4 (7431·1 to 9411·9)	2·1% (−2·6 to 7·1)
	France	4 771 184 (4 201 951 to 5 333 473)	6988·0 (6149·0 to 7847·1)	5 957 857 (5 265 047 to 6 669 257)	6988·0 (6149·4 to 7852·1)	0% (−0·1 to 0·1)
	Germany	7 112 254 (6 386 097 to 7 974 730)	6906·4 (6159·6 to 7746·1)	8 533 010 (7 527 225 to 9 540 594)	7285·2 (6385·4 to 8158·8)	5·5% (−0·6 to 16·2)
	Greece	1 622 362 (1 436 467 to 1 807 457)	12 977·0 (11 432·4 to 14 528·9)	1 859 706 (1 651 578 to 2 064 191)	12 979·3 (11 435·1 to 14 532·2)	0% (0 to 0·1)
	Iceland	14 648 (12 826 to 16 351)	5427·3 (4769·0 to 6058·5)	22 674 (19 823 to 25 357)	5517·6 (4830·7 to 6166·8)	1·7% (−2 to 6)
	Ireland	311 807 (273 616 to 350 417)	8392·5 (7332·6 to 9454·8)	489 284 (430 660 to 552 273)	8391·4 (7331·8 to 9452·7)	0% (−0·1 to 0·1)
	Israel	347 888 (305 166 to 390 141)	7310·4 (6412·1 to 8228·4)	689 296 (606 451 to 771 047)	7310·1 (6409·3 to 8227·0)	0% (−0·1 to 0·1)
	Italy	7 918 119 (7 054 657 to 8 853 357)	11 092·4 (9842·5 to 12 502·4)	9 467 762 (8 406 023 to 10 577 600)	11 093·9 (9845·7 to 12 505·6)	0% (−0·1 to 0·1)
	Luxembourg	40 379 (35 423 to 45 505)	8394·6 (7334·6 to 9458·0)	63 856 (55 952 to 72 068)	8392·2 (7331·4 to 9455·8)	0% (−0·2 to 0·1)
	Malta	34 538 (30 182 to 38 884)	8394·2 (7333·5 to 9453·4)	49 947 (43 945 to 56 224)	8393·7 (7330·8 to 9453·4)	0% (−0·1 to 0·1)
	Netherlands	1 347 045 (1 182 670 to 1 505 981)	7541·8 (6621·7 to 8447·1)	1 707 073 (1 509 099 to 1 919 211)	7540·9 (6616·1 to 8444·5)	0% (−0·1 to 0·1)
	Norway	395 050 (348 316 to 440 406)	7664·9 (6708·4 to 8676·1)	525 315 (466 938 to 587 581)	7792·1 (6863·2 to 8746·8)	1·7% (−1·7 to 4·8)
	Portugal	971 933 (861 801 to 1 090 075)	8218·3 (7255·4 to 9256·4)	1 226 572 (1 085 639 to 1 369 386)	8219·0 (7256·6 to 9258·7)	0% (0 to 0)
	Spain	3 724 906 (3 277 884 to 4 182 059)	8026·6 (7045·0 to 9004·7)	5 079 969 (4 447 388 to 5 743 612)	8028·6 (7046·7 to 9008·3)	0% (0 to 0·1)
	Sweden	1 001 950 (889 542 to 1 111 065)	9213·1 (8167·1 to 10 300·6)	1 294 507 (1 149 483 to 1 448 645)	9801·9 (8652·1 to 11 010·5)	6·4% (2·2 to 11·6)
	Switzerland	578 645 (507 769 to 648 068)	6640·9 (5820·2 to 7404·2)	771 619 (681 134 to 868 257)	6640·5 (5817·6 to 7404·1)	0% (−0·1 to 0·1)
	UK	6 889 174 (6 103 565 to 7 700 741)	9760·6 (8628·6 to 10 978·5)	8 538 870 (7 504 831 to 9 574 876)	9920·0 (8721·3 to 11 140·4)	1·6% (−1·5 to 5·1)
**Latin America and Caribbean**		**40 824 504 (35 810 139 to 46 422 081)**	**12 965·5 (11 460·2 to 14 587·4)**	**78 041 010 (69 115 477 to 87 872 082)**	**12 889·1 (11 415·1 to 14 518·8)**	**−0·6 % (−1·6 to 0·4)**
Andean Latin America		3 731 313 (3 257 871 to 4 261 040)	12 682·9 (11 167·7 to 14 258·1)	7 529 753 (6 627 259 to 8 483 887)	12 683·2 (11 166·1 to 14 259·0)	0% (0 to 0)
	Bolivia	596 208 (520 573 to 680 109)	12 682·8 (11 169·0 to 14 261·2)	1 327 781 (1 164 735 to 1 502 067)	12 683·2 (11 165·4 to 14 261·5)	0% (−0·1 to 0·1)
	Ecuador	984 351 (860 051 to 1 123 707)	12 682·9 (11 167·9 to 14 257·0)	2 062 720 (1 815 332 to 2 322 549)	12 683·2 (11 166·2 to 14 257·8)	0% (0 to 0)
	Peru	2 150 754 (1 878 290 to 2 454 276)	12 682·9 (11 167·3 to 14 257·7)	4 139 252 (3 641 224 to 4 654 106)	12 683·2 (11 166·3 to 14 258·6)	0% (0 to 0)
Caribbean		3 991 532 (3 497 824 to 4 521 623)	12 682·9 (11 168·0 to 14 259·8)	6 277 903 (5 532 059 to 7 052 480)	12 683·0 (11 167·0 to 14 257·5)	0% (0 to 0)
	Antigua and Barbuda	7149 (6291 to 8100)	12 682·4 (11 169·9 to 14 263·4)	12 936 (11 409 to 14 580)	12 683·0 (11 166·3 to 14 265·2)	0% (0 to 0·1)
	The Bahamas	28 931 (25 334 to 33 182)	12 682·6 (11 167·8 to 14 263·0)	52 211 (46 001 to 59 011)	12 682·8 (11 168·5 to 14 262·2)	0% (0 to 0)
	Barbados	34 005 (30 072 to 38 306)	12 682·6 (11 168·4 to 14 262·7)	47 091 (41 650 to 52 804)	12 682·9 (11 167·2 to 14 262·4)	0% (−0·1 to 0·1)
	Belize	16 515 (14 433 to 18 857)	12 683·3 (11 160·8 to 14 264·6)	45 096 (39 439 to 51 335)	12 683·3 (11 166·3 to 14 258·7)	0% (−0·1 to 0·1)
	Bermuda	8677 (7630 to 9794)	12 682·6 (11 166·5 to 14 258·3)	11 187 (9874 to 12 580)	12 682·2 (11 170·3 to 14 258·8)	0% (0 to 0)
	Cuba	1 430 211 (1 258 052 to 1 616 826)	12 683·4 (11 164·9 to 14 261·4)	1 844 304 (1 630 238 to 2 071 126)	12 683·6 (11 162·9 to 14 267·5)	0% (−0·1 to 0·1)
	Dominica	8456 (7464 to 9506)	12 683·2 (11 171·4 to 14 271·5)	9926 (8790 to 11 122)	12 683·4 (11 160·7 to 14 266·3)	0% (−0·3 to 0·3)
	Dominican Republic	710 149 (620 621 to 812 947)	12 682·9 (11 170·1 to 14 260·8)	1 323 819 (1 164 000 to 1 493 212)	12 683·3 (11 164·9 to 14 265·6)	0% (−0·1 to 0·1)
	Grenada	9007 (7956 to 10 158)	12 683·0 (11 168·1 to 14 263·3)	15 811 (14 042 to 17 691)	12 683·7 (11 159·9 to 14 266·6)	0% (−0·2 to 0·2)
	Guyana	77 113 (67 298 to 88 432)	12 682·9 (11 167·7 to 14 258·7)	91 162 (80 214 to 103 096)	12 682·6 (11 168·7 to 14 258·9)	0% (0 to 0)
	Haiti	593 380 (518 756 to 676 405)	12 682·4 (11 171·6 to 14 259·6)	1 284 107 (1 123 041 to 1 468 411)	12 682·6 (11 172·4 to 14 260·6)	0% (−0·1 to 0·1)
	Jamaica	252 206 (221 675 to 285 752)	12 682·5 (11 169·6 to 14 260·0)	377 583 (333 134 to 424 832)	12 682·9 (11 166·3 to 14 259·8)	0% (−0·1 to 0·1)
	Puerto Rico	463 500 (408 439 to 520 886)	12 682·7 (11 170·2 to 14 263·0)	594 065 (526 722 to 664 438)	12 682·9 (11 167·6 to 14 262·8)	0% (0 to 0·1)
	Saint Lucia	13 799 (12 100 to 15 687)	12 682·4 (11 169·2 to 14 262·4)	25 659 (22 642 to 28 869)	12 683·2 (11 164·5 to 14 259·7)	0% (−0·1 to 0·1)
	Saint Vincent and the Grenadines	11 076 (9707 to 12 598)	12 683·1 (11 165·3 to 14 265·5)	15 994 (14 110 to 18 013)	12 683·4 (11 160·9 to 14 261·2)	0% (−0·2 to 0·2)
	Suriname	42 175 (36 850 to 48 052)	12 684·1 (11 164·2 to 14 264·4)	76 193 (67 311 to 85 831)	12 682·7 (11 167·4 to 14 259·9)	0% (−0·1 to 0·1)
	Trinidad and Tobago	135 569 (118 750 to 153 892)	12 683·0 (11 164·4 to 14 260·9)	208 364 (184 208 to 235 602)	12 683·2 (11 163·7 to 14 264·3)	0% (0 to 0)
	Virgin Islands	13 028 (11 436 to 14 774)	12 682·1 (11 169·6 to 14 262·3)	16 639 (14 754 to 18 666)	12 682 (11 170·3 to 14 263·3)	0% (0 to 0)
Central Latin America		16 202 908 (14 190 997 to 18 482 230)	12 903·9 (11 430·5 to 14 518·0)	32 927 601 (29 125 287 to 37 092 301)	12 903·7 (11 433·0 to 14 518·0)	0% (−0·1 to 0·1)
	Colombia	3 383 975 (2 956 241 to 3 875 716)	12 682·7 (11 169·5 to 14 259·8)	6 718 862 (5 920 727 to 7 549 880)	12 683·2 (11 166·3 to 14 259·6)	0% (−0·1 to 0·1)
	Costa Rica	315 341 (276 125 to 361 091)	12 683·0 (11 167·2 to 14 258·9)	643 481 (567 395 to 723 408)	12 682·8 (11 168·7 to 14 263·7)	0% (−0·1 to 0·1)
	El Salvador	502 274 (438 966 to 573 898)	12 682·4 (11 172·0 to 14 261·6)	761 814 (670 024 to 860 368)	12 682·5 (11 172·8 to 14 263·6)	0% (−0·2 to 0·2)
	Guatemala	689 971 (602 229 to 788 517)	12 682·9 (11 168·8 to 14 261·8)	1 846 053 (1 614 500 to 2 104 210)	12 683·1 (11 167·5 to 14 265·5)	0% (−0·2 to 0·2)
	Honduras	393 181 (343 377 to 449 837)	12 682·8 (11 170·6 to 14 261·1)	1 007 462 (880 458 to 1 148 430)	12 682·6 (11 172·4 to 14 260·5)	0% (−0·1 to 0·1)
	Mexico	8 452 831 (7 387 586 to 9 644 548)	13 119·0 (11 540·7 to 14 739·9)	16 678 596 (14 662 553 to 18 784 601)	13 119·3 (11 540·8 to 14 741·3)	0% (0 to 0)
	Nicaragua	319 114 (278 058 to 366 614)	12 682·5 (11 171·2 to 14 261·2)	737 071 (645 613 to 835 402)	12 683·3 (11 166·5 to 14 261·5)	0% (−0·1 to 0·1)
	Panama	255 097 (223 058 to 290 541)	12 683·3 (11 163·7 to 14 262·3)	505 780 (445 649 to 567 933)	12 683·3 (11 164·9 to 14 261·5)	0% (0 to 0)
	Venezuela	1 891 125 (1 651 827 to 2 167 886)	12 682·9 (11 166·9 to 14 258·1)	4 028 482 (3 541 208 to 4 537 112)	12 682·9 (11 166·3 to 14 260·8)	0% (0 to 0)
Tropical Latin America		16 898 751 (14 716 683 to 19 258 614)	13 152·4 (11 596·9 to 14 827·0)	31 305 753 (27 526 387 to 35 334 144)	12 958·9 (11 418·2 to 14 600·6)	−1·5% (−3·9 to 1)
	Brazil	16 499 682 (14 360 444 to 18 802 424)	13 155·4 (11 591·9 to 14 830·9)	30 463 453 (26 754 226 to 34 342 971)	12 955·6 (11 418·2 to 14 595·0)	−1·5% (−4 to 1)
	Paraguay	399 068 (349 892 to 455 320)	13 021·6 (11 466·4 to 14 678·1)	842 300 (740 428 to 955 897)	13 021·6 (11 469·0 to 14 677·8)	0% (0 to 0)
**North Africa and Middle East**		**30 409 759 (26 651 332 to 34 720 257)**	**11 977·8 (10 573·2 to 13 498·2)**	**68 737 046 (60 245 631 to 77 878 368)**	**12 058·5 (10 664·8 to 13 566·8)**	**0·7 % (−0·2 to 1·7)**
	Afghanistan	866 025 (760 034 to 981 040)	11 910·9 (10 512 to 13 437·5)	2 484 705 (2 163 607 to 2 871 704)	11 894·7 (10 498·6 to 13 420·9)	−0·1% (−0·4 to 0·1)
	Algeria	2 171 467 (1 896 799 to 2 490 045)	11 895·3 (10 498·2 to 13 416·2)	4 877 477 (4 275 335 to 5 548 813)	11 897·1 (10 496·8 to 13 418·7)	0% (−0·1 to 0·1)
	Bahrain	54 061 (46 587 to 63 372)	11 824 (10 429·3 to 13 359·5)	204 219 (175 514 to 236 172)	11 813·5 (10 429·0 to 13 338·9)	−0·1% (−0·3 to 0·1)
	Egypt	5 089 584 (4 450 614 to 5 805 974)	11 895·4 (10 495·4 to 13 417·5)	10 119 891 (8 879 145 to 11 523 085)	11 891·0 (10 490·6 to 13 413·9)	0% (−0·1 to 0)
	Iran	5 001 253 (4 348 508 to 5 710 069)	12 351·3 (10 840·8 to 13 889·6)	11 052 904 (9 649 638 to 12 561 238)	12 365·1 (10 862·4 to 13 918·4)	0·1% (0 to 0·2)
	Iraq	1 400 026 (1 223 677 to 1 596 509)	11 887·5 (10 490·0 to 13 411·5)	4 177 739 (3 652 431 to 4 762 436)	11 888·3 (10 489·5 to 13 412·2)	0% (0 to 0)
	Jordan	274 157 (237 764 to 313 949)	10 863·8 (9576·4 to 12 192·1)	1 011 184 (884 296 to 1 144 168)	10 821·9 (9541·8 to 12 151·1)	−0·4% (−0·5 to −0·2)
	Kuwait	189 295 (163 778 to 220 860)	11 833·1 (10 436·8 to 13 354·4)	577 936 (500 561 to 671 152)	11 875·4 (10 465·1 to 13 408·0)	0·4% (0 to 0·8)
	Lebanon	365 936 (321 117 to 417 478)	11 904·5 (10 509·1 to 13 430·1)	952 420 (834 858 to 1 080 846)	11 892·0 (10 501·6 to 13 415·9)	−0·1% (−0·2 to 0)
	Libya	353 363 (308 680 to 405 034)	11 864·1 (10 461·7 to 13 397·2)	815 503 (711 147 to 932 910)	11 888·0 (10 489·5 to 13 412·2)	0·2% (0 to 0·4)
	Morocco	2 381 645 (2 088 381 to 2 713 632)	11 901·6 (10 503·0 to 13 424·4)	4 308 151 (3 786 260 to 4 880 340)	11 899·2 (10 500·9 to 13 420·9)	0% (−0·1 to 0)
	Palestine	155 922 (135 793 to 178 800)	11 906·3 (10 510·5 to 13 427·8)	458 141 (399 597 to 522 602)	11 891·5 (10 493·0 to 13 412·0)	−0·1% (−0·3 to 0·1)
	Oman	169 120 (146 831 to 196 152)	11 810·6 (10 419·8 to 13 351·2)	577 981 (495 229 to 679 229)	11 785·3 (10 394·9 to 13 324·9)	−0·2% (−0·5 to 0·1)
	Qatar	50 998 (43 680 to 60 075)	11 772·7 (10 386·4 to 13 315·1)	388 768 (331 774 to 457 340)	11 753·4 (10 348·7 to 13 278·7)	−0·2% (−0·5 to 0·1)
	Saudi Arabia	1 606 058 (1 389 297 to 1 853 815)	13 599·5 (11 975·6 to 15 268·6)	5 270 480 (4 597 349 to 5 998 352)	14 034·5 (12 526·4 to 15 665·6)	3·2% (−1·8 to 10·4)
	Sudan	1 667 811 (1 457 905 to 1 902 618)	11 904·5 (10 504·0 to 13 427·8)	3 537 829 (3 090 727 to 4 047 773)	11 908·6 (10 508·7 to 13 435·8)	0% (0 to 0·1)
	Syria	992 553 (865 064 to 1 138 813)	11 894·2 (10 492·7 to 13 415·9)	1 869 431 (1 636 743 to 2 120 229)	11 895·5 (10 497·1 to 13 421·9)	0% (−0·1 to 0·1)
	Tunisia	812 012 (714 143 to 925 180)	11 900·3 (10 500·5 to 13 420·9)	1 507 001 (1 323 918 to 1 709 880)	11 902·3 (10 505·6 to 13 427·1)	0% (−0·1 to 0·1)
	Turkey	5 610 409 (4 920 408 to 6 379 722)	11 627·3 (10 285·5 to 13 120·0)	10 364 102 (9 185 978 to 11 704 150)	11 705·2 (10 382·5 to 13 157·0)	0·7% (−3·3 to 4·7)
	United Arab Emirates	202 916 (173 302 to 239 297)	11 774·0 (10 395·0 to 13 313·3)	1 483 707 (1 244 676 to 1 769 798)	11 760·9 (10 364·4 to 13 305·7)	−0·1% (−0·4 to 0·2)
	Yemen	975 545 (852 148 to 1 115 838)	11 897·0 (10 500·7 to 13 424·8)	2 633 288 (2 296 767 to 3 017 753)	11 897·6 (10 502·7 to 13 421·6)	0% (−0·1 to 0·1)
**South Asia**		**65 242 270 (56 902 371 to 73 929 511)**	**7780·9 (6845·7 to 8745·2)**	**125 641 877 (109 974 457 to 142 166 532)**	**7626·3 (6704·6 to 8564·8)**	**−2·0 % (−4·8 to 1·2)**
	Bangladesh	5 883 280 (5 086 564 to 6 746 810)	8100·8 (7073·5 to 9132·3)	11 929 522 (10 392 617 to 13 546 219)	8104·5 (7076·1 to 9139·6)	0% (−0·2 to 0·2)
	Bhutan	30 145 (26 154 to 34 504)	7717·0 (6782·7 to 8645·0)	68 868 (59 673 to 78 903)	7717·2 (6788·5 to 8643·6)	0% (−0·2 to 0·2)
	India	52 482 902 (45 724 767 to 59 502 412)	7751·7 (6794·8 to 8704·0)	98 695 062 (86 507 971 to 111 721 091)	7555·9 (6648·5 to 8490·2)	−2·5% (−6 to 1·5)
	Nepal	1 057 570 (919 017 to 1 199 947)	7716·3 (6781·0 to 8647·2)	2 035 175 (1 777 734 to 2 291 534)	7715·6 (6776·5 to 8659·5)	0% (−0·2 to 0·2)
	Pakistan	5 788 373 (5 050 852 to 6 549 723)	7717·2 (6782·2 to 8645·8)	12 913 251 (11 219 751 to 14 697 558)	7716·8 (6780·1 to 8644·7)	0% (−0·1 to 0·1)
**Southeast Asia, east Asia, and Oceania**		**108 018 549 (93 904 101 to 123 102 854)**	**6825·5 (5978·0 to 7643·8)**	**175 090 522 (153 095 489 to 196 638 213)**	**6848·2 (5992·8 to 7681·8)**	**0·3 % (0 to 0·6)**
East Asia		78 509 585 (68 321 208 to 89 300 084)	6548·5 (5723·4 to 7358·9)	121 776 731 (106 423 295 to 136 940 451)	6531·2 (5700·2 to 7332·8)	−0·3% (−0·6 to 0·1)
	China	74 123 021 (64 440 571 to 84 473 388)	6511·4·0 (5696·4 to 7313·5)	115 053 108 (100 589 950 to 129 393 020)	6484·9 (5659·4 to 7281·1)	−0·4% (−0·7 to −0·1)
	North Korea	1 415 675 (1 238 275 to 1 608 965)	7095·0 (6239·9 to 7998·1)	2 153 622 (1 897 013 to 2 433 689)	7101·7 (6250·6 to 8005·9)	0·1% (−0·1 to 0·3)
	Taiwan (Province of China)	1 662 905 (1 464 259 to 1 884 376)	8115·6 (7194·6 to 9126·7)	2 608 247 (2 283 337 to 2 932 327)	8557·3 (7514·6 to 9662·3)	5·4% (0 to 11·8)
Oceania		363 844 (315 044 to 418 897)	7565·4 (6656·8 to 8524·6)	781 383 (678 823 to 900 548)	7564·5 (6658 to 8522·7)	0% (−0·1 to 0)
	American Samoa	2848 (2468 to 3275)	7566·0 (6655·3 to 8523·2)	3832 (3358 to 4350)	7562·3 (6654·6 to 8519·9)	0% (−0·2 to 0·1)
	Federated States of Micronesia	5448 (4728 to 6267)	7564·8 (6650·7 to 8526·3)	6976 (6093 to 7957)	7564·0 (6651·0 to 8525·9)	0% (−0·1 to 0·1)
	Fiji	46 294 (40 191 to 53 357)	7563·1 (6655·2 to 8521·8)	66 752 (58 641 to 75 723)	7564·2 (6651·8 to 8524·7)	0% (−0·1 to 0·1)
	Guam	9465 (8186 to 10 881)	7569·1 (6659·0 to 8520·8)	13 224 (11 640 to 14 893)	7565·8 (6654·3 to 8525·6)	0% (−0·2 to 0·1)
	Kiribati	4365 (3779 to 5028)	7560·7 (6647·8 to 8519·0)	7432 (6477 to 8549)	7559·6 (6647·3 to 8518·6)	0% (−0·1 to 0·1)
	Marshall Islands	2189 (1897 to 2538)	7566 (6654·9 to 8525·7)	3703 (3223 to 4236)	7564·6 (6654·2 to 8525·0)	0% (−0·1 to 0·1)
	Northern Mariana Islands	3243 (2802 to 3794)	7575·6 (6674·5 to 8533·2)	3886 (3370 to 4411)	7566·0 (6658·7 to 8522·9)	−0·1% (−0·6 to 0·4)
	Papua New Guinea	226 468 (195 742 to 261 081)	7565·9 (6657·5 to 8525·1)	558 468 (484 269 to 645 395)	7564·8 (6661·1 to 8522·5)	0% (−0·1 to 0·1)
	Samoa	9074 (7886 to 10 375)	7564·7 (6656·4 to 8525·5)	12 365 (10 812 to 14 042)	7566·0 (6655·0 to 8527·0)	0% (−0·1 to 0·1)
	Solomon Islands	16 956 (14 723 to 19 509)	7565·6 (6664·8 to 8522·9)	37 445 (32 526 to 43 119)	7563·5 (6657·0 to 8521·2)	0% (−0·2 to 0·1)
	Tonga	5435 (4753 to 6172)	7559·4 (6657·5 to 8515·0)	6848 (5997 to 7756)	7562·8 (6651·5 to 8522·3)	0% (−0·1 to 0·2)
	Vanuatu	8052 (6979 to 9281)	7565·6 (6662·7 to 8521·0)	17 392 (15 184 to 19 896)	7563·4 (6659·0 to 8519·7)	0% (−0·1 to 0·1)
Southeast Asia		29 145 120 (25 325 828 to 33 365 900)	7717·0 (6796·7 to 8706·4)	52 532 409 (46 065 854 to 59 422 836)	7712·7 (6793·6 to 8700·6)	−0·1% (−0·1 to 0)
	Cambodia	534 682 (462 237 to 614 523)	7553·6 (6648·7 to 8505·4)	1 123 132 (983 024 to 1 282 230)	7559·2 (6646·0 to 8518·3)	0·1% (−0·1 to 0·3)
	Indonesia	11 878 970 (10 303 277 to 13 633 368)	7956·5 (7005·7 to 9006·1)	20 837 890 (18 259 685 to 23 735 645)	7958·1 (7005·4 to 9012·3)	0% (0 to 0·1)
	Laos	223 941 (195 171 to 256 072)	7559·6 (6653·4 to 8520·1)	465 243 (405 810 to 534 147)	7562·5 (6652·6 to 8521·8)	0% (−0·1 to 0·1)
	Malaysia	1 080 504 (939 298 to 1 241 568)	7563·1 (6652·8 to 8521·5)	2 364 050 (2 071 205 to 2 680 115)	7565·5 (6653·2 to 8527·0)	0% (−0·1 to 0·1)
	Maldives	10 930 (9474 to 12 543)	7566·0 (6668·9 to 8522·2)	36 954 (31 918 to 42 835)	7577·6 (6671·9 to 8522·2)	0·2% (−0·4 to 0·7)
	Mauritius	77 469 (67 487 to 88 573)	7562·3 (6649·3 to 8522·8)	115 778 (102 035 to 130 232)	7562·8 (6650·1 to 8522·4)	0% (0 to 0)
	Myanmar	2 477 119 (2 154 301 to 2 835 551)	7561·0 (6651·8 to 8519·0)	3 990 303 (3 507 305 to 4 511 431)	7558·5 (6649·1 to 8518·1)	0% (−0·1 to 0·1)
	Philippines	3 609 380 (3 125 032 to 4 145 211)	7562·5 (6652·3 to 8521·3)	7 098 353 (6 201 755 to 8 086 339)	7563·8 (6651·8 to 8524·9)	0% (−0·1 to 0·1)
	Sri Lanka	1 155 324 (1 007 932 to 1 323 357)	7562·8 (6657·2 to 8520·9)	1 790 553 (1 577 425 to 2 016 351)	7559·8 (6650·1 to 8518·9)	0% (−0·2 to 0·1)
	Seychelles	4939 (4324 to 5613)	7563·9 (6651·9 to 8526·9)	8579 (7540 to 9696)	7568·6 (6659·3 to 8515·2)	0·1% (−0·1 to 0·3)
	Thailand	3 951 064 (3 442 819 to 4 537 422)	7561·1 (6651·9 to 8519·3)	6 677 997 (5 863 268 to 7 514 335)	7561·1 (6650·8 to 8520·0)	0% (0 to 0)
	East Timor	42 549 (36 717 to 49 364)	7564·8 (6657·3 to 8523·7)	75 927 (66 294 to 86 448)	7564·2 (6655·3 to 8520·6)	0% (−0·1 to 0·1)
	Vietnam	4 059 573 (3 530 963 to 4 655 972)	7557·3 (6648·0 to 8514·3)	7 878 528 (6 924 407 to 8 906 273)	7562·0 (6650·4 to 8522·4)	0·1% (−0·1 to 0·2)
**Sub-Saharan Africa**		**33 082 466 (28 818 435 to 37 656 551)**	**10 082·2 (8863·2 to 11 288·7)**	**72 356 270 (62 992 341 to 82 538 296)**	**10 079·7 (8858·7 to 11 285)**	**0 % (−0·1 to 0·1)**
Central sub-Saharan Africa		3 624 034 (3 147 653 to 4 133 745)	9972·7 (8796·0 to 11 158·3)	8 414 692 (7 315 758 to 9 603 190)	9973·1 (8798·2 to 11 164·9)	0% (−0·1 to 0·1)
	Angola	675 466 (586 963 to 772 268)	9975·4 (8798·9 to 11 167·8)	1 832 038 (1 590 905 to 2 092 591)	9972·0 (8797·3 to 11 161·0)	0% (−0·3 to 0·2)
	Central African Republic	185 824 (161 138 to 212 145)	9972·5 (8796·5 to 11 157·3)	343 986 (298 610 to 391 890)	9974·5 (8801·0 to 11 170·4)	0% (−0·1 to 0·2)
	Congo (Brazzaville)	163 056 (141 551 to 186 013)	9971·8 (8796·7 to 11 161·5)	395 815 (344 838 to 449 769)	9974·9 (8800·5 to 11 173·7)	0% (−0·1 to 0·2)
	Democratic Republic of the Congo	2 499 114 (2 171 223 to 2 848 156)	9972·2 (8794·9 to 11 155·4)	5 606 505 (4 872 721 to 6 397 172)	9973·3 (8798·2 to 11 165·3)	0% (−0·2 to 0·2)
	Equatorial Guinea	27 965 (24 295 to 31 543)	9971·1 (8795·0 to 11 159·4)	91 065 (78 663 to 105 171)	9971·5 (8790·9 to 11 152·7)	0% (−0·3 to 0·3)
	Gabon	72 609 (63 313 to 82 253)	9972·2 (8800·8 to 11 160·8)	145 282 (126 613 to 164 030)	9973·8 (8797·8 to 11 165·4)	0% (−0·2 to 0·2)
Eastern sub-Saharan Africa		12 294 679 (10 677 029 to 14 040 881)	10 149·9 (8918·8 to 11 356·8)	26 995 872 (23 475 580 to 30 950 777)	10 154·5 (8922·3 to 11 359·7)	0% (0 to 0·1)
	Burundi	355 257 (308 934 to 406 605)	9971·1 (8796·5 to 11 156·8)	726 783 (631 557 to 832 364)	9975·8 (8801·6 to 11 170·8)	0% (−0·2 to 0·3)
	Comoros	30 327 (26 321 to 34 544)	9973·9 (8796·4 to 11 164·3)	60 321 (52 823 to 68 115)	9973·4 (8798·7 to 11 166·0)	0% (−0·1 to 0·1)
	Djibouti	32 391 (27 845 to 37 423)	9976·8 (8797·0 to 11 168·0)	95 574 (83 037 to 108 764)	9978·6 (8798·7 to 11 176·5)	0% (−0·1 to 0·1)
	Eritrea	178 175 (154 456 to 204 191)	9970·7 (8794·7 to 11 163·3)	422 713 (366 965 to 484 648)	9970·2 (8796·9 to 11 170·8)	0% (−0·1 to 0·1)
	Ethiopia	3 341 820 (2 901 979 to 3 832 667)	10 440·1 (9163·5 to 11 746·2)	7 124 884 (6 166 813 to 8 190 584)	10 441·0 (9166·6 to 11 752·5)	0% (−0·1 to 0·1)
	Kenya	1 456 981 (1 262 317 to 1 676 273)	10 439·6 (9163·9 to 11 747·1)	3 691 026 (3 203 570 to 4 233 788)	10 439·1 (9165·1 to 11 743·9)	0% (−0·1 to 0·1)
	Madagascar	786 364 (683 591 to 898 263)	9974·7 (8797·1 to 11 161·7)	1 796 418 (1 560 475 to 2 049 172)	9974·2 (8796·9 to 11 164·4)	0% (−0·1 to 0·1)
	Malawi	633 617 (549 868 to 723 735)	9973·0 (8797·8 to 11 163·2)	1 181 087 (1 023 830 to 1 353 242)	9971·8 (8799·5 to 11 164·0)	0% (−0·1 to 0·1)
	Mozambique	951 201 (827 690 to 1 079 289)	9972·7 (8796·4 to 11 162·1)	1 927 155 (1 672 081 to 2 205 982)	9971·2 (8798·3 to 11 162·1)	0% (−0·1 to 0·1)
	Rwanda	465 665 (404 986 to 532 620)	9971·2 (8795·6 to 11 153·2)	919 720 (800 241 to 1 048 303)	9970·6 (8794·9 to 11 162·5)	0% (−0·1 to 0·1)
	Somalia	451 740 (393 552 to 514 181)	9975·4 (8797·0 to 11 165·3)	1 091 117 (942 357 to 1 247 368)	9974·7 (8798·7 to 11 166·6)	0% (−0·1 to 0·1)
	South Sudan	393 252 (340 660 to 450 545)	9979·8 (8794·8 to 11 175·9)	636 345 (554 832 to 723 458)	9976·7 (8797·2 to 11 170·9)	0% (−0·3 to 0·2)
	Tanzania	1 665 221 (1 447 060 to 1 899 224)	9973·2 (8797·8 to 11 161·4)	3 727 015 (3 242 389 to 4 240 066)	9973·2 (8797·6 to 11 162·4)	0% (0 to 0)
	Uganda	1 057 285 (917 646 to 1 210 058)	9974·5 (8798·0 to 11 165·0)	2 412 090 (2 094 201 to 2 768 812)	9971·8 (8797·2 to 11 162·1)	0% (−0·2 to 0·1)
	Zambia	489 142 (423 790 to 559 858)	9974·7 (8797·4 to 11 162·1)	1 166 683 (1 012 343 to 1 341 276)	9974·4 (8801·3 to 11 171·2)	0% (−0·2 to 0·2)
Southern sub-Saharan Africa		4 112 764 (3 593 418 to 4 667 510)	10 324·2 (9078·3 to 11 573·0)	7 399 095 (6 452 697 to 8 370 632)	10 335·4 (9091·3 to 11 600·9)	0·1% (0 to 0·2)
	Botswana	88 663 (77 044 to 101 153)	9971·4 (8797·7 to 11 162·3)	207 814 (181 017 to 236 379)	9971·7 (8798·9 to 11 159·4)	0% (−0·1 to 0·1)
	eSwatini	49 919 (43 314 to 56 986)	9971·8 (8797·4 to 11 167·2)	88 455 (76 785 to 100 993)	9970·7 (8798·9 to 11 166·6)	0% (−0·2 to 0·1)
	Lesotho	130 771 (113 912 to 147 890)	9972·4 (8799·7 to 11 165·6)	166 106 (144 803 to 188 488)	9970·1 (8798·3 to 11 171·3)	0% (−0·2 to 0·1)
	Namibia	100 196 (87 218 to 113 895)	9972·8 (8798·0 to 11 166·1)	195 667 (170 669 to 222 106)	9971·0 (8797·0 to 11 160·2)	0% (−0·1 to 0·1)
	South Africa	3 084 326 (2 682 135 to 3 515 508)	10 436·6 (9158·4 to 11 742·3)	5 661 666 (4 926 049 to 6 408 656)	10 436·6 (9161·3 to 11 744·1)	0% (−0·1 to 0·1)
	Zimbabwe	658 888 (571 916 to 751 708)	9973·9 (8797·7 to 11 165·5)	1 079 387 (937 165 to 1 229 132)	9970·3 (8799·4 to 11 160·6)	0% (−0·3 to 0·2)
Western sub-Saharan Africa		13 050 989 (11 368 184 to 14 815 672)	9976·5 (8801·8 to 11 172·1)	29 546 610 (25 702 505 to 33 703 585)	9973·1 (8798·1 to 11 161·8)	0% (−0·2 to 0·2)
	Benin	300 081 (260 686 to 342 519)	9972·1 (8797·3 to 11 161·3)	777 255 (675 035 to 889 233)	9973·1 (8796·7 to 11 164·4)	0% (−0·1 to 0·2)
	Burkina Faso	601 123 (523 411 to 679 021)	9971·9 (8795·6 to 11 159·6)	1 402 749 (1 219 498 to 1 598 034)	9971·6 (8796·3 to 11 158·7)	0% (−0·1 to 0·1)
	Cameroon	683 204 (593 984 to 777 587)	9973·6 (8798·5 to 11 164·4)	1 936 150 (1 683 850 to 2 219 153)	9974·2 (8798·8 to 11 165·8)	0% (−0·1 to 0·1)
	Cape Verde	24 593 (21 535 to 27 766)	9967·6 (8788·4 to 11 147·9)	52 259 (45 749 to 58 867)	9973·4 (8799·3 to 11 166·3)	0·1% (−0·4 to 0·5)
	Chad	386 159 (336 683 to 438 001)	9972·0 (8795·8 to 11 158·3)	890 697 (772 885 to 1 017 758)	9976·1 (8797·5 to 11 169·2)	0% (−0·2 to 0·3)
	Côte d'Ivoire	781 980 (676 623 to 897 574)	9977·2 (8801·3 to 11 171·4)	1 822 656 (1 584 068 to 2 081 403)	9977·0 (8802·7 to 11 170·9)	0% (−0·1 to 0)
	The Gambia	62 614 (54 223 to 71 820)	9977·4 (8802·4 to 11 176·3)	150 031 (130 181 to 171 968)	9974·4 (8798·1 to 11 167·9)	0% (−0·2 to 0·2)
	Ghana	1 018 657 (884 046 to 1 160 600)	9974·1 (8799·0 to 11 167·2)	2 404 418 (2 091 578 to 2 732 648)	9971·5 (8797·0 to 11 158·9)	0% (−0·2 to 0·1)
	Guinea	427 909 (373 725 to 482 825)	9974 (8797·1 to 11 166·3)	803 402 (699 187 to 915 324)	9973·6 (8796·2 to 11 163·7)	0% (−0·1 to 0)
	Guinea-Bissau	63 239 (54 944 to 72 147)	9972·3 (8794·9 to 11 157·2)	127 114 (110 065 to 145 966)	9972·2 (8795·9 to 11 160·6)	0% (−0·1 to 0·1)
	Liberia	137 277 (120 722 to 154 523)	9977·1 (8803·0 to 11 174·4)	339 532 (295 574 to 387 042)	9976·1 (8800·0 to 11 171·8)	0% (−0·1 to 0·1)
	Mali	571 001 (496 150 to 645 898)	9974·1 (8798·4 to 11 165·6)	1 281 127 (1 113 166 to 1 461 137)	9975·3 (8799·2 to 11 169·7)	0% (−0·1 to 0·1)
	Mauritania	141 578 (123 338 to 160 981)	9973·5 (8797·6 to 11 163·3)	283 014 (246 873 to 320 695)	9973·8 (8796·4 to 11 162·0)	0% (−0·1 to 0·1)
	Niger	481 801 (418 426 to 549 732)	9975·9 (8799·5 to 11 171·2)	1 224 640 (1 061 994 to 1 398 476)	9973·6 (8795·2 to 11 160·0)	0% (−0·2 to 0·1)
	Nigeria	6 382 642 (5 562 220 to 7 242 068)	9979·0 (8790·3 to 11 164·7)	13 848 607 (12 058 033 to 15 784 151)	9972·2 (8798·2 to 11 158·8)	−0·1% (−0·5 to 0·3)
	São Tomé and Príncipe	8049 (7025 to 9092)	9973·0 (8794·9 to 11 162·3)	15 560 (13 560 to 17 633)	9974 (8798·9 to 11 164·6)	0% (−0·1 to 0·1)
	Senegal	485 760 (422 155 to 553 320)	9973·9 (8797·1 to 11 162·1)	1 062 457 (925 166 to 1 205 532)	9973·4 (8795·9 to 11 160·5)	0% (−0·1 to 0·1)
	Sierra Leone	268 422 (234 385 to 304 939)	9974·6 (8798·7 to 11 167·0)	561 252 (487 108 to 642 976)	9975·3 (8799·1 to 11 170·7)	0% (−0·1 to 0·1)
	Togo	224 534 (194 807 to 257 389)	9972·3 (8793·9 to 11 157·5)	563 399 (489 556 to 640 241)	9972 (8796·9 to 11 165·7)	0% (−0·2 to 0·1)

The super-regions North Africa and the Middle East and South Asia each contain only one region, which bears the same name, so these rows are not repeated. 95% UI=95% uncertainty interval.

**Figure 2:**
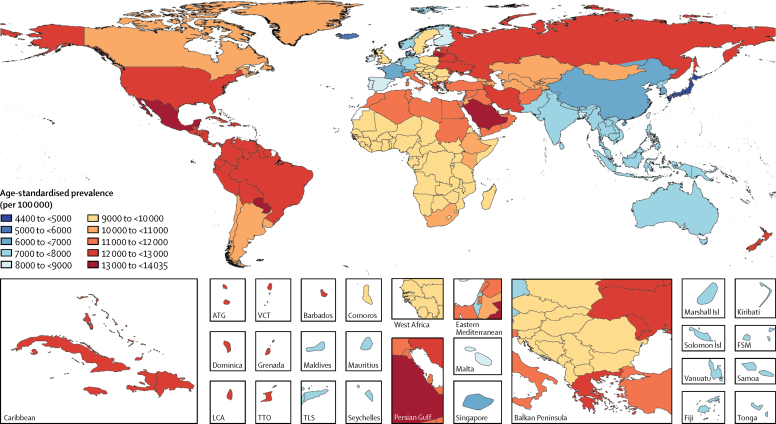
Age-standardised prevalence (per 100 000 population) of gastro-oesophageal reflux disease, for both sexes, in 2017 ATG=Antigua and Barbuda. VCT=Saint Vincent and the Grenadines. LCA=Saint Lucia. TTO=Trinidad and Tobago. Isl=Islands. FSM=Federated States of Micronesia. TLS=Timor-Leste.

**Figure 3:**
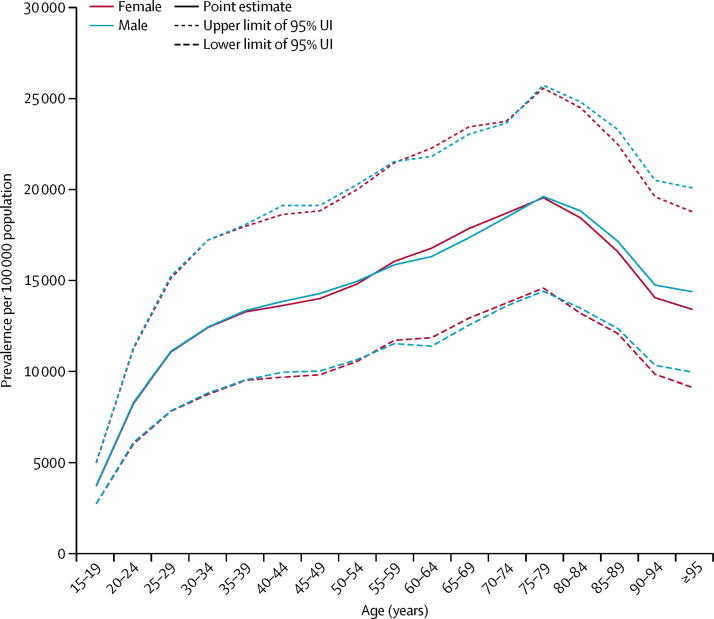
Prevalence (per 100 000 population) of gastro-oesophageal reflux disease, by age group, in 2017 95% UI=95% uncertainty interval.

Mean estimates of age-standardised prevalence of gastro-oesophageal reflux disease for all locations in 2017 ranged from 4408 per 100 000 population in Japan to 14 035 cases per 100 000 population in Saudi Arabia (table). Geographical variation in the age-standardised prevalence of gastro-oesophageal reflux disease in 2017 is shown in figure 2. Standardised for age, gastro-oesophageal reflux disease was most prevalent in the USA, Italy, Greece, New Zealand, and several countries in Latin America and the Caribbean (excluding southern Latin America), north Africa and the Middle East, and eastern Europe, at more than 11 000 cases per 100 000 population. Age-standardised prevalence was lowest in high-income Asia Pacific, east Asia, Iceland, France, Denmark, and Switzerland, at less than 7000 cases per 100 000 population. The ratio of age-standardised prevalence among males versus females was 1·0 globally in both 1990 and 2017, ranging from 0·98 to 1·00 across super-regions. Prevalence increased with age, peaking at age 75–79 years overall and for both sexes (18 820 [95% UI 13 770–24 000] cases per 100 000 population for both sexes combined; illustrated for each sex separately in figure 3).

The global age-standardised prevalence of gastro-oesophageal reflux disease was stable over time, at 8791 (95% UI 7772 to 9834) cases per 100 000 population in 1990 and 8819 (7781 to 9863) cases per 100 000 population in 2017, with a percentage change of 0·3% (−0·3 to 0·9). The percentage change in age-standardised prevalence was also small, with an uncertainty interval that includes zero for all GBD regions except for high-income North America, where estimates increased by 5·4% (1·7 to 9·3), high-income Asia Pacific, where estimates increased by 6·8% (4·2 to 10·5), and Australasia, where estimates decreased by 0·6% (0·5 to 0·8). Without age standardisation, however, global all-age prevalence increased by 18·1% (15·6 to 20·4) between 1990 and 2017, from 7859 (6905 to 8851) cases per 100 000 population in 1990 to 9283 (8189 to 10 400) cases per 100 000 population in 2017. A larger increase between 1990 and 2017 was seen in the global count of prevalent cases—from 424 million (372 to 477) in 1990 to 709 million (626 to 795) in 2017, a change of 67·2% (63·8 to 70·6).

**Figure 4:**
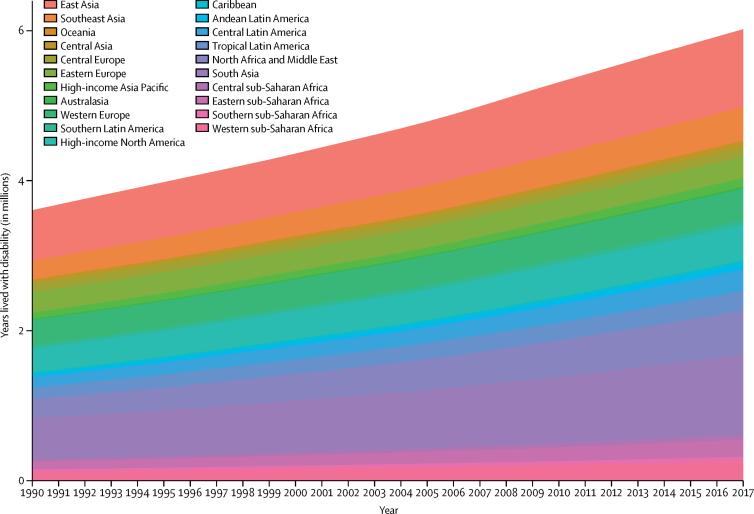
Years lived with disability due to gastro-oesophageal reflux disease for all GBD regions, 1990–2017 GBD=Global Burden of Diseases, Injuries, and Risk Factors Study.

YLDs due to gastro-oesophageal reflux disease for all locations estimated in GBD 2017 are shown in the table in the appendix (pp 12–20). Globally, gastro-oesophageal reflux disease was responsible for 3·60 million (95% UI 1·93–6·12) YLDs in 1990. By 2017, this had increased to 6·01 million (3·22–10·19), an increase of 67·1% (63·5–70·3). In 1990, gastro-oesophageal reflux disease was responsible for 0·6% (0·4–1·0) of all YLDs globally, and in 2017 it was responsible for 0·7% (0·4–1·1) of all YLDs globally, which represents a 10·1% (7·4–12·6) increase in relative contribution to non-fatal health loss. This can be compared with the YLDs contributed to global non-fatal health loss by other conditions by use of a tree map on the GBD Compare Online Hub. An increase in total YLDs over time was seen across all GBD regions, as shown in figure 4.

All-age YLD rates also increased globally, from 67 (95% UI 36 to 113) per 100 000 population in 1990 to 79 (42 to 133) per 100 000 population in 2017, an increase of 18·0% (15·5 to 20·2), and increased in each GBD super-region (data not shown). Age-standardised YLD rates, however, remained stable across that period, at 74 (40 to 126) per 100 000 population in 1990 and 75 (40 to 127) per 100 000 population in 2017, representing a 0·6% (−0·2 to 1·2) change. As seen for age-standardised prevalence, age-standardised YLD rates did not change significantly between 1990 and 2017 in most GBD regions; exceptions were high-income North America (5·3% [1·7 to 9·3]), high-income Asia Pacific (6·8% [4·1 to 10·6]), and eastern sub-Saharan Africa (0·7% [0·2 to 1·1]).

Geographical variation in age-standardised YLD rates reflects variation in prevalence. YLD rates by age also reflect variation in prevalence, with a peak rate at ages 75–79 years globally (appendix p 10). No relationship was seen between age-standardised gastro-oesophageal reflux disease YLD rate and Socio-demographic Index (appendix p 11).

## Discussion

We estimated a global increase in total YLDs due to gastro-oesophageal reflux disease between 1990 and 2017, and in YLD rates in populations, but stable YLD rates when standardised to a reference age distribution. This discrepancy between a stable age-standardised YLD rate but rising all-age YLD rate over time reflects higher prevalence in older age groups and the ageing of the global population over time.^53^ Age-standardised prevalence of gastro-oesophageal reflux disease is estimated to be highest in the USA, Italy, New Zealand, and countries in Latin America and the Caribbean (excluding southern Latin America), north Africa and the Middle East, and eastern Europe, and lowest in high-income Asia Pacific, east Asia, and some countries in western Europe. In contrast to the global trend and most other regions, high-income North America and high-income Asia Pacific showed increases in the age-standardised YLD rate due to gastro-oesophageal reflux disease between 1990 and 2017. In these regions there could be factors contributing to increasing gastro-oesophageal reflux disease burden beyond just demographic changes. However, additional factors contributing to the changing burden in these two regions and factors associated with spatial variation in gastro-oesophageal reflux disease prevalence were not identified here. The fact that established risk factors of high BMI, alcohol, and smoking were not predictive in our model raises the question of whether spatial and temporal variation in these results is driven more by measurement error than by underlying epidemiology.

Our results are largely consistent with previous systematic reviews and one meta-analysis of gastro-oesophageal reflux disease, reporting prevalence estimates ranging from approximately 10% to 30% in the USA and the Middle East and from 3% to 8% in east Asian countries.^4^, ^38^, ^39^ Our regional estimates are similar to the regional pooled estimates in the meta-analysis of Eusebi and colleagues,^4^ with higher estimates in the Americas and the Middle East, and lower estimates for Asia, although GBD 2017 estimates were generally lower than Eusebi and colleagues' estimates for near-equivalent geographies. A noteworthy difference is that Eusebi and colleagues estimated very high prevalence in South Asia, 22·1% (95% CI 11·5–35·0), well above the GBD estimate of 7·0% (95% UI 6·2–8·0), but very similar estimates in southeast Asia (7·4% *vs* 8·1%).^4^ These differences are likely to be due to the fact that Eusebi and colleagues did not adjust for variations in study design; more than half of the 106 studies included in Eusebi and colleagues' regional estimates had at least one study design characteristic that would have prompted adjustment in the GBD 2017 modelling approach.^4^ These differences are consistent with Eusebi and colleagues' finding of a lower global prevalence estimate based on only a subset of studies that met a more stringent case definition.^4^ The systematic review by El-Serag and colleagues^39^ also noted higher estimates of gastro-oesophageal reflux disease prevalence for studies published in 1995–2009 compared to studies published before 1995, although it did not report a temporal difference over time for studies published after 1995. Similarly, GBD 2017 prevalence estimates rose between 1990 and 2017, but this rise is largely attenuated with age standardisation, which is not addressed by El-Serag and colleagues. Eusebi and colleagues did not test temporal trends.

Our analysis has several limitations. The first and most important limitation is scarce input data and absence of data for many locations. Prevalence data for modelling gastro-oesophageal reflux disease total 144 location-years, similar to many chronic diseases, such as migraine headache (124 location-years of prevalence data), but substantially lower than better-studied diseases such as diabetes (2340 location-years of prevalence data).^42^ Scarce data restrict the precision of estimates for all locations. The absence of data for particular locations requires estimates for those locations to be determined by regional, super-regional, and global estimates. Our estimation of YLDs from prevalence data is also limited by scarce data about the distribution of symptom severity and frequency, and the resulting assumption that these distributions are the same across years, age groups, sexes, and locations. Additional data will be sought in future rounds of GBD, and additional population-based studies of gastro-oesophageal reflux disease prevalence, severity, and symptom frequency should be done, particularly in locations with few or no data.

A second data limitation is that input studies use heterogeneous study designs and are subject to potential biases that are only partially overcome in the DisMod modelling framework. Estimating fixed effects for study design characteristics in successive mixed-effects models essentially corrects for potential study-design biases on the basis of ecological comparisons, and cannot fully adjust for variation in study design if certain designs are preferentially used in some years and locations more than others. In future rounds, we should use pre-modelling adjustments for bias that use internal comparisons of case definitions from validation studies or inter-study comparisons of design features between studies that are well matched in location and time. With additional data and improved pre-modelling data adjustments, associations between gastro-oesophageal reflux disease prevalence data and established risk factors such as high BMI, obesity, and smoking should be re-evaluated, to see whether they can further strengthen predictions in data-sparse locations. Since data on gastro-oesophageal reflux disease are taken primarily from surveys, sometimes with low response rates, that were focused on gastrointestinal symptoms and potentially influenced by commercial interest, future rounds of GBD should seek data from general household surveys with high response rates, and consider adjustments to data from surveys that announce a focus on gastrointestinal symptoms (which might bias participation), have poor response rates, or are commercially sponsored.

A third limitation is that our case definition required an individual to have typical reflux symptoms at least weekly for 12 months. This definition is consistent with a published meta-analysis^4^ and similar to expert group recommendations for population-based research on gastro-oesophageal reflux disease,^1^ but might miss individuals who have appreciable symptoms over shorter periods of time, those who have atypical symptoms, and those who have asymptomatic mucosal injury and risk of complications. Future rounds of GBD should estimate burden due to these additional presentations of the disease. Conversely, symptom-based definitions might include individuals with similar symptoms not due to reflux of stomach contents, such as those with functional dyspepsia. Differences might exist in the association between symptoms and findings on diagnostic studies by location. Validation studies in representative populations should be done to estimate the predictive value of symptom-based questionnaires compared to more comprehensive and specific case definitions.

Finally, health loss due to conditions for which gastro-oesophageal reflux disease is a risk factor (such as oesophageal carcinoma) is accounted for in separate GBD estimates, but the relationship to gastro-oesophageal reflux disease should be made more explicit in future rounds to fully account for the effect of this disease on human health.

Our study has several strengths. We have incorporated more prevalence data sources than previously published systematic reviews and one previous meta-analysis. More importantly, GBD 2017 is, to our knowledge, the first study to apply methods of meta-regression to estimate the prevalence of gastro-oesophageal reflux disease, which offers several advantages. Rather than qualitatively assessing the differences in study design that might explain differences in estimates of epidemiological measures from diverse sources, we have accounted quantitatively for many of these important differences using fixed effects for study-level covariates. Rather than reporting estimates only for age groups, years, and locations for which prevalence data have been collected, we have generated estimates for all age groups, years, and locations, incorporating information from adjacent age groups, years, and locations to calculate the best possible prevalence estimates where no data are available. Although estimates for locations without data are less certain, they provide policy makers and other stakeholders with the best available knowledge about the possible extent of this problem, and a tool by which to gauge the value of further research on this disease relative to expenditures in other areas.

The choice of time period for GBD, 1990–2017, also offers the chance to observe trends in gastro-oesophageal reflux disease epidemiology during a period of increasing obesity prevalence. An association between obesity and gastro-oesophageal reflux disease has been observed in previous studies,^2^, ^3^, ^4^ suggesting that gastro-oesophageal reflux disease might rise in the 1990–2017 period. The fact that we did not see a rise in age-standardised prevalence of gastro-oesophageal reflux disease in this period does not undermine the association reported in these studies, which were done at the individual level, and could be due to data or modelling limitations (as discussed previously); it could also imply the existence of other risk factors with a large influence on global gastro-oesophageal reflux disease occurrence.

Finally, GBD 2017 is the first study to move beyond measuring gastro-oesophageal reflux disease occurrence to estimating the relative burden that gastro-oesophageal reflux disease imposes in terms of YLDs, facilitating comparison with the burden of other diseases and injuries.

In conclusion, GBD 2017 identifies gastro-oesophageal reflux disease as an important cause of non-fatal health loss, which is increasing because of its association with age and the ageing of the global population. Our estimates also show an increase in prevalence after age standardisation for some locations, but variation in age-standardised prevalence was not associated with known risk factors and might be due to measurement error we could not adjust for with current data and methods. These findings indicate that health-care systems need to be prepared to address the needs of increasing numbers of patients with gastro-oesophageal reflux disease. Further studies are needed to identify useful public health interventions. Given the costs and adverse outcomes associated with symptomatic treatment for gastro-oesophageal reflux disease (such as pulmonary infection and loss of bone-mineral density associated with long-term proton-pump inhibitor use) and the increased risk of oesophageal carcinoma in people with gastro-oesophageal reflux disease, additional large, high-quality studies of gastro-oesophageal reflux disease prevalence are needed to verify these findings. Further research is required to identify more modifiable risk factors for gastro-oesophageal reflux disease, and to develop more effective interventions to modify its established risk factors and its relationship to oesophageal carcinoma.^54^

## Data sharing

To download the data used in these analyses, please visit the Global Health Data Exchange.
