# Supplementary material for: The global, regional, and national burden of gastro-oesophageal reflux disease in 195 countries and territories, 1990–2017: a systematic analysis for the Global Burden of Disease Study 2017
Source: Lancet Gastroenterol Hepatol. 2020 Mar 13;5(6):561–81. doi: 10.1016/S2468-1253(19)30408-X (PMC7232025; doi:10.1016/S2468-1253(19)30408-X)
Supplement: Supplementary appendix [file mmc1.pdf]

THE LANCET
Gastroenterology & Hepatology

Supplementary appendix

This appendix formed part of the original submission and has been peer reviewed. We post it as supplied by the authors.

Supplement to: GBD 2017 Gastro-oesophageal Reflux Disease Collaborators. The global, regional, and national burden of gastro-oesophageal reflux disease in 195 countries and territories, 1990–2017: a systematic analysis for the Global Burden of Disease Study 2017. *Lancet Gastroenterol Hepatol* 2020; published online April 13. https://doi.org/10.1016/S2468-1253(19)30408-X.

Methods Appendix, "The global, regional, and national burden of gastro-oesophageal reflux disease in 195 countries and territories, 1990–2017: a systematic analysis for the Global Burden of Disease Study 2017"

Modelling flowsheet for estimation of non-fatal health loss due to gastro-oesophageal reflux disease (GORD), GBD 2017

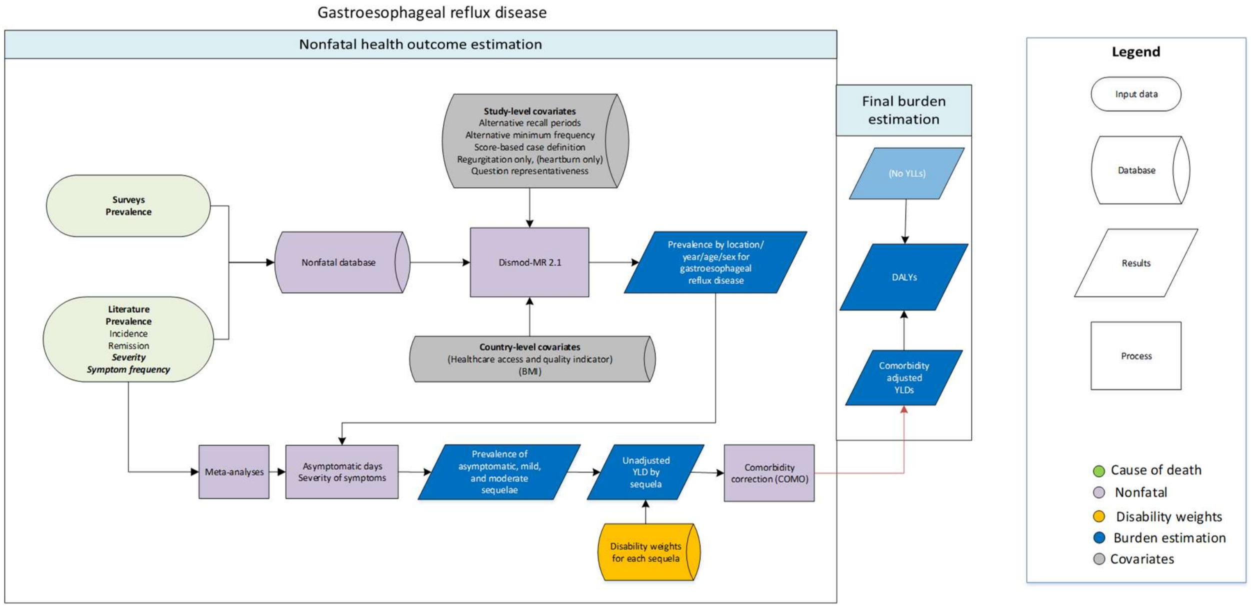

Search terms, inclusion and exclusion criteria for data sources for modelling gastro-oesophageal reflux disease (GORD), GBD 2017

PubMed search terms:

("gastroesophageal reflux disease"[MeSH Terms]
OR "heartburn"[MeSH Terms]
OR "GERD"[Title/Abstract]
OR "gastroesophageal reflux disease"[Title/Abstract]
OR "heartburn"[Title/Abstract]
OR "GORD"[Title/Abstract]
OR "GER"[Title/Abstract]
OR "acid reflux"[Title/Abstract]
OR "barett's esophagus"[Title/Abstract]
OR "reflux disease"[Title/Abstract]
OR "hiatal hernia"[Title/Abstract]
OR ("achalasia"[Title/Abstract] AND "cardia"[Title/Abstract])

OR (
("gastroesophageal"[Title/Abstract] OR "gastro oesophageal"[Title/Abstract] OR "esophagus"[Title/Abstract] OR "esophageal"[Title/Abstract])
AND ("hernia"[Title/Abstract] OR "ulcer"[Title/Abstract] OR "obstruction"[Title/Abstract] OR "perforation"[Title/Abstract] OR "laceration"[Title/Abstract] OR "dyskinesia"[Title/Abstract] OR "diverticulum"[Title/Abstract])
)

AND ("prevalence"[Title/Abstract] OR "incidence"[Title/Abstract)
AND ("1990/01/01"[PDAT] : "2017/07/26"[PDAT])
NOT(animals[MeSH] NOT humans[MeSH])

)

Studies were included if cases were identified from a general population defined only by location and years of observation, or the source population was arguably representative of such a population, and results included one or more estimates of prevalence, incidence, or remission of GORD. Due to diverse case definitions encountered, studies that defined cases based on symptom-based questionnaires, self-reported diagnosis, diagnostic codes in facilities or claims data, or using one of several diagnostic tests (pH manometry, EGD, trial of empiric therapy) were all included, but studies had to include sufficient information on case definition and diagnostics to identify those needing adjustment to our standard case-definition.

Studies that were conducted in a clinically or demographically defined subpopulation or were otherwise non-representative, or where source population was not adequately described, were excluded. Studies that did not provide sufficient uncertainty information were excluded (percentage prevalence reported without sample size, standard error, or confidence interval). All studies of incidence and remission provided inadequate information on person-time of observation and loss to follow-up, and so they were excluded. Only publications in English, Spanish, French, and Portuguese could be extracted by our research team; studies reported in other languages were not included due to lack of translation. Modelling studies and meta-analyses were not included as input data.

The USA National Health Interview Survey in 2007 and 2012 asked participants about the occurrence of typical reflux symptoms. This survey was identified from the Global Health Data Exchange (GHDx) and extracted by the GORD modeler but met the same inclusion and exclusion criteria applied to the published studies identified by our PubMed search.

Flowsheet of systematic review for data sources for modelling gastro-oesophageal reflux disease (GORD), GBD 2017

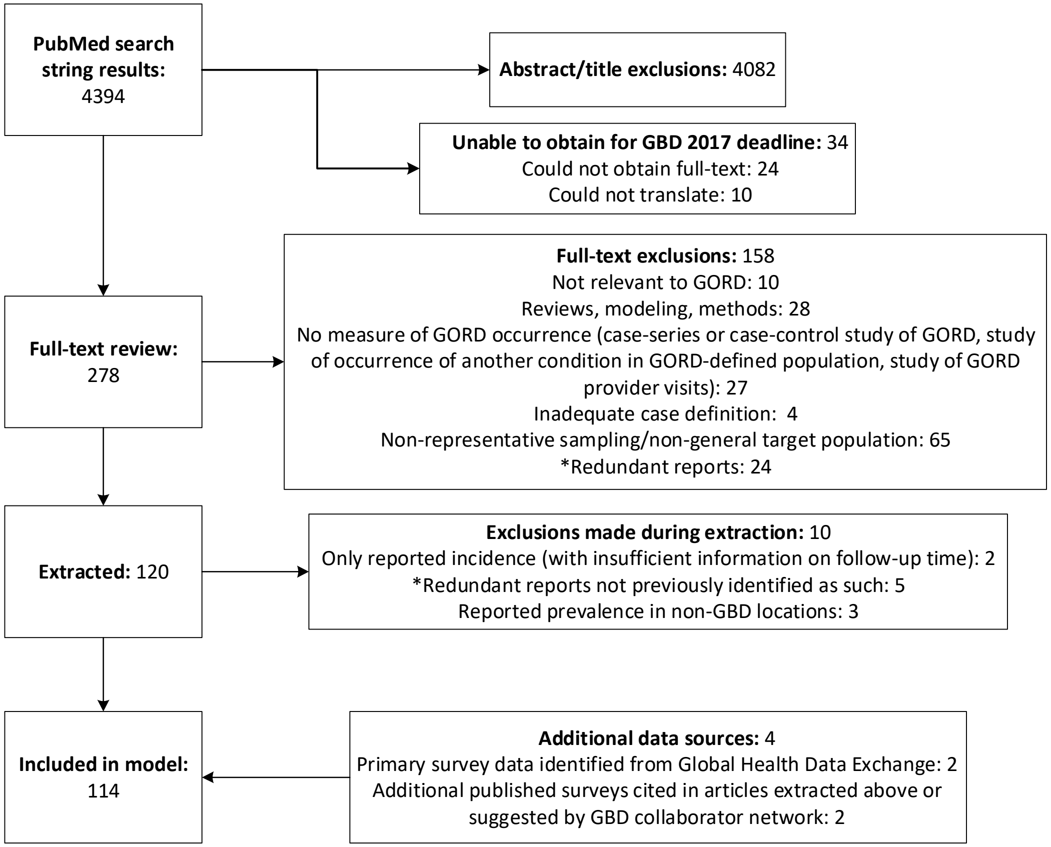

***If redundant reports were found, information may have been extracted from more than one report (to get complete and detailed methods, age-sex-distribution, severity distribution, etcetera), but the source is counted only once in the total sources included in the model, with one or more publications counted among the exclusions.**

Studies found in the systematic review may have reported prevalence for multiple locations or years. Our database ultimately included 144 location-years of data; “location-years” are unique combinations of source, year and location, which GBD publications report to more accurately describe data availability. Data stratified by age and sex, although preferable for modelling, are not distinguished from both-sex, all-age data in these counts.

Study-level covariates in DisMod-MR 2.1 meta-regression for gastro-oesophageal reflux disease (GORD), GBD 2017

Covariate	Coefficients	Exponentiated coefficients
Diagnostic score	0.66 (0.41 to 0.91)	1.94 (1.51 to 2.48)
Regurgitation only	- 0.62 (- 0.93 to - 0.31)	0.54 (0.39 to 0.73)
Minimum monthly	0.22 (- 0.12 to 0.57)	1.24 (0.89 to 1.78)
No minimum	0.57 (0.32 to 0.83)	1.76 (1.37 to 2.30)
1-month recall	- 0.65 (- 0.96 to - 0.35)	0.52 (0.38 to 0.70)
3-month recall	- 0.3 (- 0.55 to - 0.066)	0.74 (0.58 to 0.94)
Recall not specified	0.087 (- 0.13 to 0.28)	1.09 (0.88 to 1.32)
Non-representative sample	0.19 (- 0.014 to 0.39)	1.21 (0.99 to 1.48)
General survey	- 0.39 (- 0.79 to - 0.036)	0.67 (0.45 to 0.97)

Location-level covariates tried and not found to be predictive include mean BMI, cumulative cigarette exposure, per capita alcohol consumption, Healthcare Access and Quality Index

Assumptions in estimating years lived with disability (YLDs) for gastro-oesophageal reflux disease (GORD), GBD 2017

The following key assumptions were made to estimate YLDs from prevalence for GORD.

First, we assumed that different levels of severity of GORD symptoms that were presented to participants in different studies could be validly mapped to lay descriptions of health states for which quantitative disability weights have been previously estimated (see references 45-48 of main text). Because our lay description of heartburn and reflux did not specify interference with daily activities or not (see “Lay description” column of table below for full wording), and in the above-cited series of surveys we found participant responses to various health states to be significantly influenced by language about interference with daily activities, we chose to map all severity levels that stated symptoms did not interfere with daily activity levels to a health state that also specifies no interference. For this, we chose our generic description of a mild abdominal problem. We mapped greater severity levels to our description of heartburn and reflux. We assumed words such as “mild” and “moderate” described symptoms that did not interfere, and words such as “severe” described symptoms that did interfere. Examples of how different scales were mapped are shown in the table below.

Severity levels in published reports, mapped to GBD severity levels, health states, and disability weights, with proportions

<table>
<tr><td colspan="6">Literature severity scales (examples)</td><td>GBD severity</td><td>Proportion</td><td>Lay description</td><td>Disability weight</td><td>Proportion</td></tr>
<tr><td rowspan="2">Mild: can be ignored</td><td>Very mild</td><td rowspan="2">Mild: don’t feel it if I don’t think of it</td><td rowspan="3">Mild: did not interfere with daily activities</td><td rowspan="3">Non-severe</td><td rowspan="3">Mild: no effect on daily life</td><td rowspan="3">Mild/moderate</td><td rowspan="3">0.72
(0.71 – 0.74)</td><td rowspan="3">Has pain in the belly that causes nausea but does not interfere with daily activities.</td><td rowspan="2">Asymptomatic days: None</td><td rowspan="2">0.42
(0.38 – 0.46)</td></tr>
<tr><td>Mild</td></tr>
<tr><td>Moderate: cannot be ignored but does not affect lifestyle</td><td>Moderate</td><td>Moderate: feel it but it doesn’t disturb my life/work</td><td>Symptomatic days:
0.011 (0.005–0.021)</td><td>0.58
(0.54 – 0.62)</td></tr>
<tr><td>Severe: affects lifestyle</td><td>Moderately severe</td><td rowspan="2">Severe: totally disturbing</td><td>Moderate: caused some disturbance in daily activities</td><td rowspan="2">Severe</td><td>Moderate: slight effect on daily life</td><td rowspan="2">Severe/very severe</td><td rowspan="2">0.28
(0.26 – 0.29)</td><td rowspan="2">Often has a burning sensation in the back of the chest after eating</td><td>Asymptomatic days: None</td><td>0.42
(0.38 – 0.46)</td></tr>
<tr><td>Very severe: has marked effect on lifestyle/ affects lifestyle “a lot”</td><td>Severe</td><td>Severe: markedly affected life and/or led to absenteeism from work</td><td>Severe: marked/considerable/strong effect on daily life</td><td>Symptomatic days:
0.027
(0.015 - 0.046)</td><td>0.58
(0.54 – 0.62)</td></tr>
</table>

The proportions of prevalent cases in the severe/very severe GBD severity level from each study (after mapping study-specific severity levels to GBD severity levels) are shown in the following forest plot, with the meta-analysis result below.

Meta-analysis of proportion severe/very severe for gastro-oesophageal reflux disease (GORD), GBD 2017

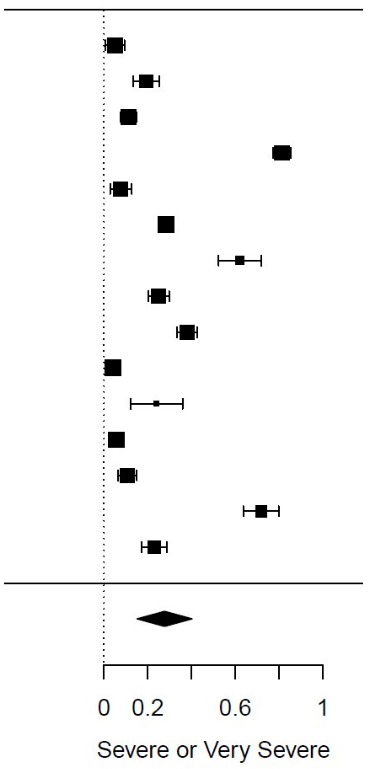

The final factor in calculating YLDs is the time spent symptomatic. We estimated the distribution of days/week spent symptomatic for cases in each study in our database that reported detailed symptom frequency information for all cases. These generally reported the proportion of cases that experienced symptoms at a given frequency. For studies with frequency categories corresponding to 1d/week, 2d/week, 3d/week, 4d/week, 5d/week, 6d/week, and daily, we could directly calculate the weighted average number of days spent symptomatic by all cases by weighting each day by the proportion of cases in that category. For studies with fewer groups (daily, several days/week and once a week, for example), we assumed a uniform distribution within the categories and used the mid-point of the frequency category as the mean for the group (in the example above, assumed mean days symptomatic of 7d/week, 4d/week, and 1d/week, and weighted these by the proportion of cases in each group to get the mean days symptomatic for all cases).

The mean number of days/week with symptoms for each study, after the calculations above, are shown in the forest plot below, along with the meta-analysis result.

Meta-analysis of days/week spent symptomatic for gastro-oesophageal reflux disease (GORD), GBD 2017

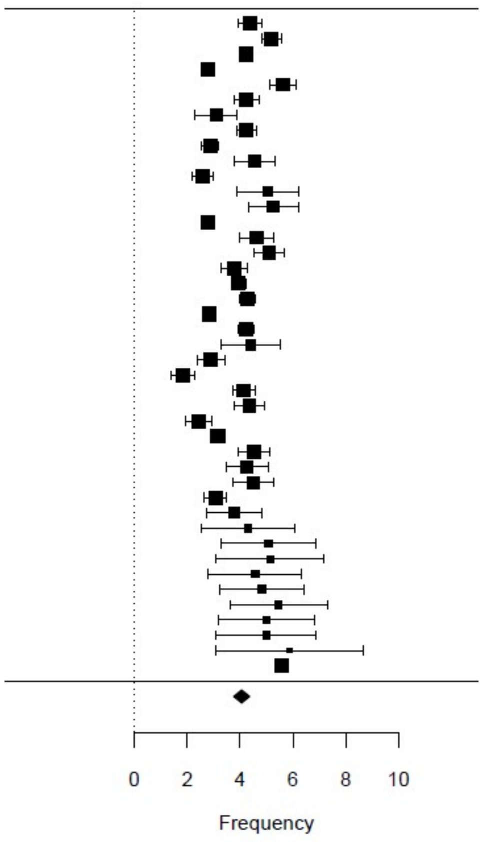

Some additional assumptions in the above process is that the frequency with which GORD cases experience symptoms, and the proportions with mild/mod vs severe/very severe symptoms are independent, the same across all year-age-sex-location combinations, regardless of prevalence, and adequately described by the subset of population-based prevalence studies in our database that explicitly report frequency and severity distributions among cases.

Although all steps in estimating YLDs are carried out using the prevalence estimates for each combination of year, age, sex and location, and are calculated using 1000 draws of a bootstrap distribution for all the following quantities, the simple calculations shown here illustrate the mechanics of YLD estimation at the global level.

Global count of prevalent cases in 2017 ~ 700 million
Proportion symptomatic (point, based on mean 4d/week symptomatic) ~ 0.58
Proportion of symptomatic severe/very severe ~ 0.28
Proportion of symptomatic mild/moderate ~ 0.72

Age-standardised rate of YLDs due to GORD, both sexes, 2017

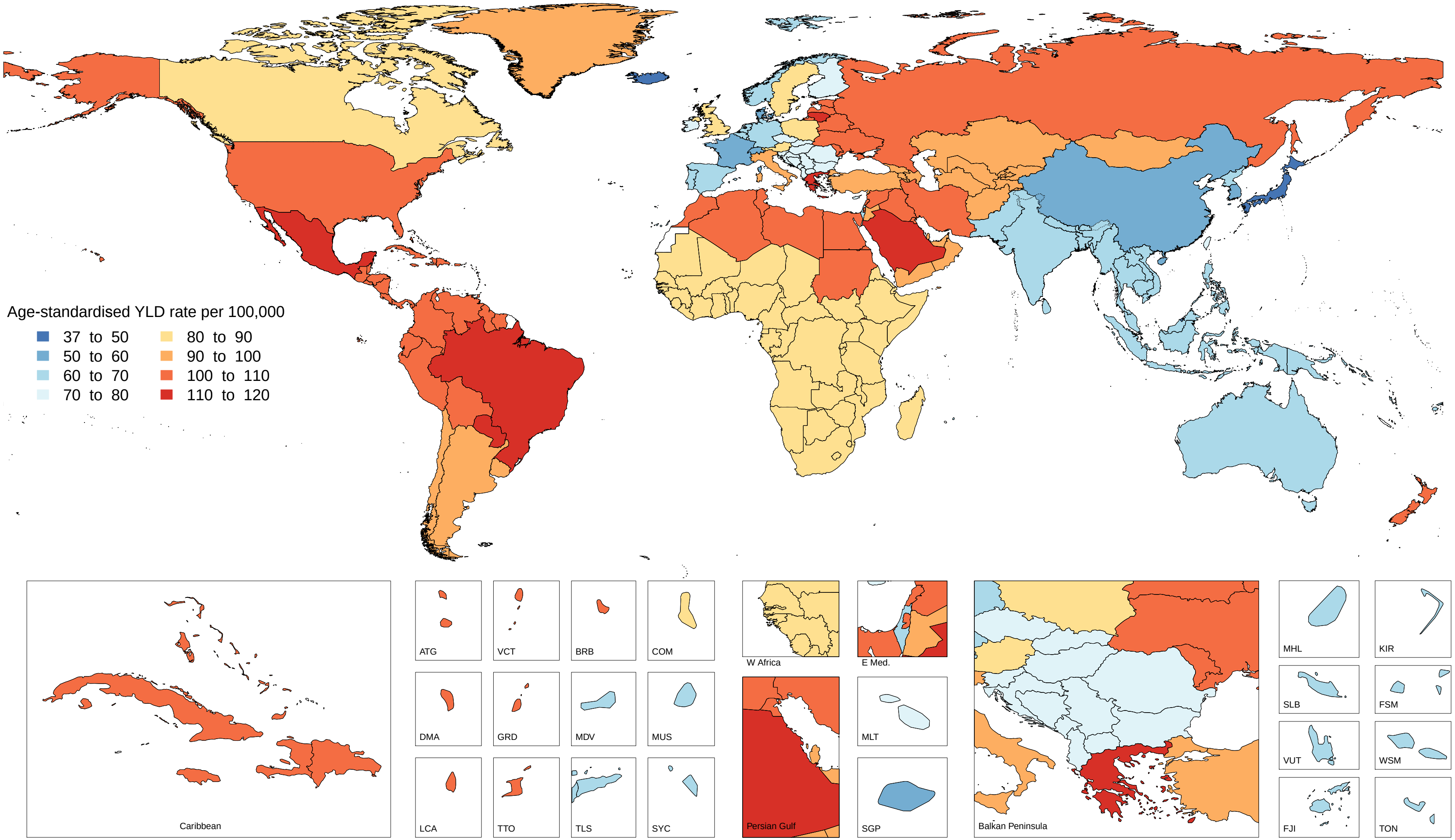

Global GORD YLD rate by age for males and females in 2017

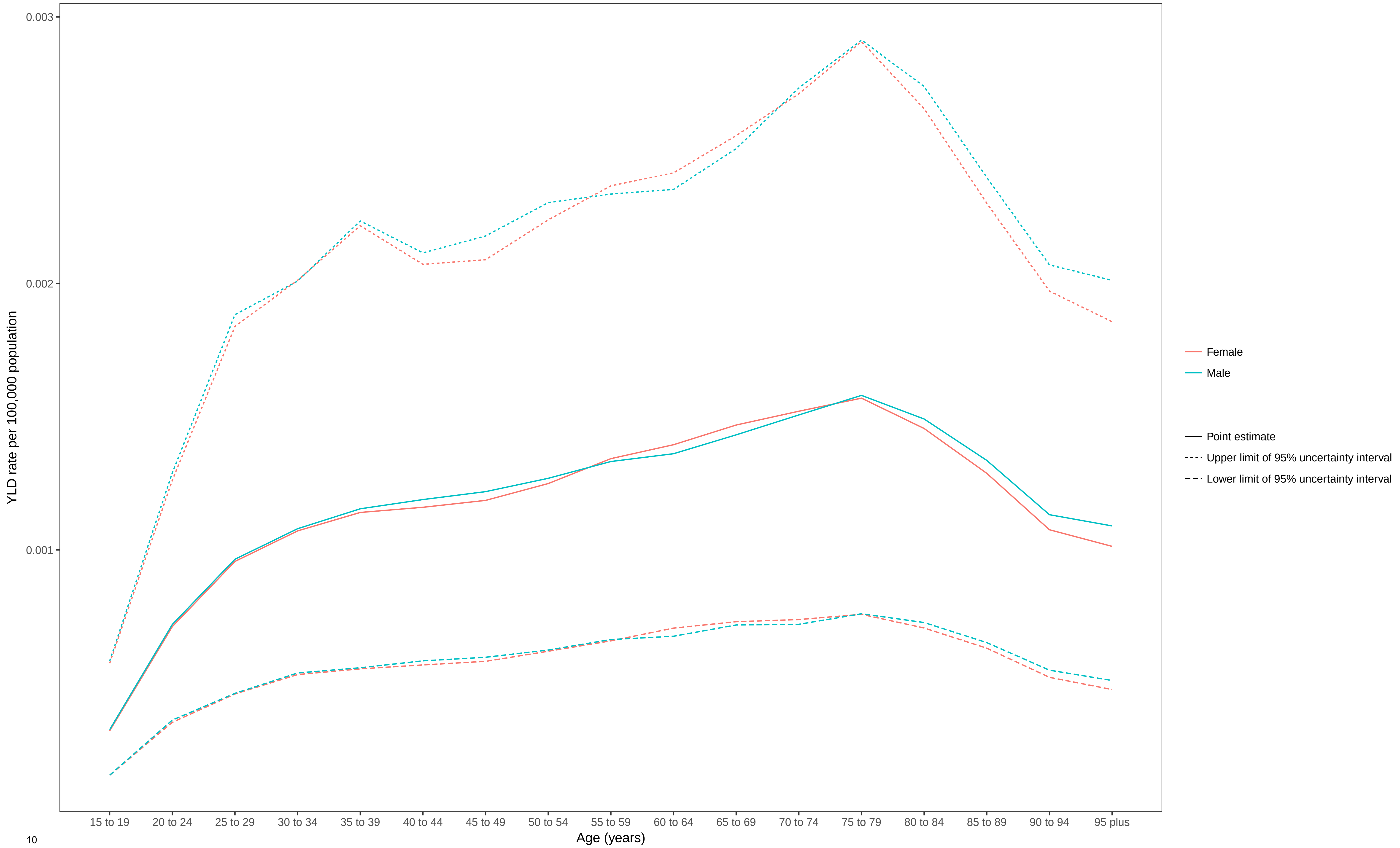

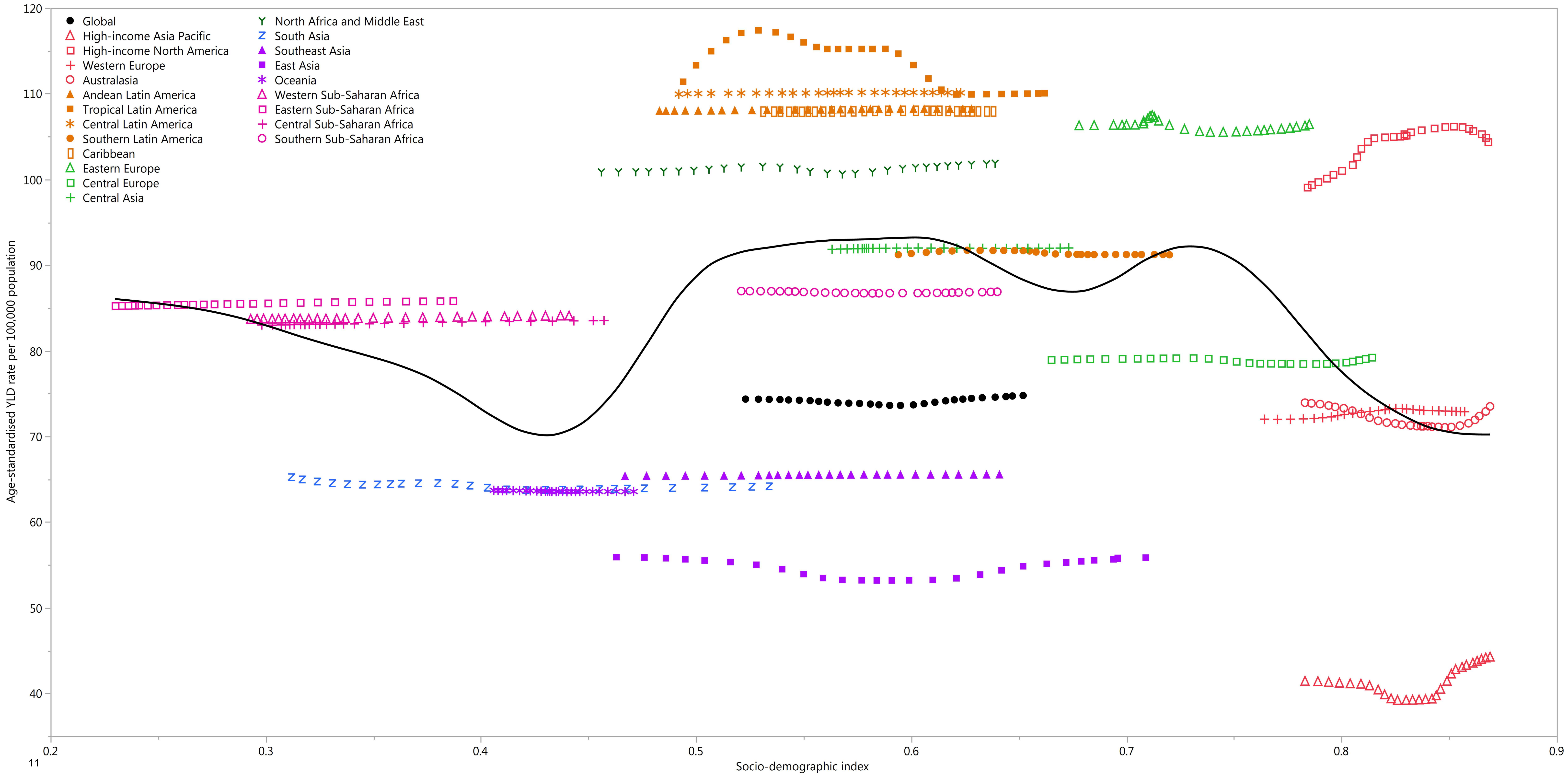
Global
High-income Asia Pacific
High-income North America
Western Europe
Australasia
Andean Latin America
Tropical Latin America
Central Latin America
Southern Latin America
Caribbean
Eastern Europe
Central Europe
Central Asia
North Africa and Middle East
South Asia
Southeast Asia
East Asia
Oceania
Western Sub-Saharan Africa
Eastern Sub-Saharan Africa
Central Sub-Saharan Africa
Southern Sub-Saharan Africa
Age-standardised YLD rate per 100,000 population
Socio-demographic index
120
110
100
90
80
70
60
50
40
0.2
0.3
0.4
0.5
0.6
0.7
0.8
0.9

YLDs due to gastro-oesophageal reflux disease in 1990 and 2017 for both sexes and all locations, with percentage change					
	1990		2017		Percentage change in age-standardised YLD rate between 1990 and 2017
	Counts (95% UI)	Age-standardised rates (95% UI)	Counts (95% UI)	Age-standardised rates (95% UI)	
**Global**	**3599710 (1930148, 6121035)**	**74.3 (39.8, 126)**	**6013380 (3215907, 10192883)**	**74.8 (40.1, 126.9)**	**0.6 (-0.2, 1.2)**
**Central Europe, Eastern Europe, and Central Asia**	**438107 (236249, 742751)**	**96.1 (51.4, 162.4)**	**491699 (263251, 830290)**	**95.8 (51.4, 162.2)**	**-0.3 (-0.6, 0.1)**
Central Asia	***54045 (28753, 92944)***	***91.9 (48.9, 156.7)***	***83289 (44675, 141986)***	***92 (49.1, 157.2)***	***0.2 (-0.5, 0.8)***
Armenia	2991 (1604, 5115)	91.9 (48.9, 156.3)	3348 (1787, 5695)	92.1 (49.3, 157.1)	0.2 (-1.2, 1.6)
Azerbaijan	5939 (3167, 10178)	92.1 (48.8, 157.1)	10216 (5447, 17340)	92.1 (49.2, 156.3)	0 (-1.3, 1.4)
Georgia	5495 (2924, 9392)	92.2 (49.1, 157.4)	4200 (2225, 7152)	92.1 (49.1, 157.3)	-0.1 (-1.4, 1.1)
Kazakhstan	14306 (7656, 24346)	91.7 (48.8, 155.9)	17168 (9173, 29218)	92 (49, 156.8)	0.3 (-1.1, 1.8)
Kyrgyzstan	3362 (1798, 5789)	91.8 (48.9, 156.4)	5376 (2842, 9151)	92.1 (49.1, 157.3)	0.4 (-0.9, 1.7)
Mongolia	1431 (765, 2455)	92 (48.9, 157.5)	2944 (1573, 4987)	92 (48.3, 156.1)	0 (-1.5, 1.4)
Tajikistan	3528 (1879, 6068)	92 (48.9, 157.1)	7200 (3813, 12342)	92.1 (49, 157.9)	0.1 (-1.3, 1.4)
Turkmenistan	2553 (1361, 4403)	92.1 (48.6, 156.8)	4497 (2419, 7684)	92.3 (49.2, 158.1)	0.2 (-1.2, 1.6)
Uzbekistan	14439 (7704, 24891)	91.9 (48.9, 156.5)	28339 (15098, 48446)	92 (49.1, 157.6)	0.2 (-1.3, 1.5)
Central Europe	***109176 (58502, 182604)***	***79 (42, 132.8)***	***120503 (64262, 201322)***	***79.2 (42.2, 133.7)***	***0.4 (-0.3, 1)***
Albania	2215 (1177, 3775)	78.4 (41.8, 134.7)	2607 (1398, 4365)	77.6 (41.8, 132)	-1 (-5.3, 3.1)
Bosnia and Herzegovina	3720 (1984, 6317)	78.3 (41.8, 133.1)	3503 (1870, 5866)	78.1 (42.1, 131.8)	-0.2 (-1.7, 1.5)
Bulgaria	8386 (4494, 14014)	78.3 (42, 132.3)	7638 (4033, 12853)	78.5 (41.9, 132.8)	0.2 (-1.1, 1.5)
Croatia	4556 (2428, 7678)	78.3 (41.5, 132.3)	4524 (2397, 7659)	78.5 (41.6, 133.3)	0.2 (-1.3, 1.7)
Czech Republic	9299 (4973, 15532)	78.1 (41.6, 131.4)	11100 (5898, 18595)	78.2 (41.8, 132.2)	0.2 (-1.5, 1.8)
Hungary	9450 (5071, 15957)	75.8 (40.4, 128.6)	10001 (5384, 16816)	76.1 (40.8, 130.4)	0.5 (-1.2, 2.2)
Republic of North Macedonia	1622 (872, 2761)	78.4 (42.1, 132.9)	2182 (1161, 3679)	78.5 (41.8, 133.4)	0.1 (-1.3, 1.5)
Montenegro	514 (275, 866)	78.4 (41.9, 131.8)	612 (327, 1033)	78.4 (41.7, 132.6)	0 (-1.6, 1.6)

Poland	34634<br>(18439, 58941)	81.4<br>(43.6, 137.8)	41433<br>(22125, 69566)	81.6<br>(43.3, 138)	0.2<br>(-1.2, 1.7)
Romania	20142<br>(10756, 34101)	78<br>(41.9, 132.2)	20201<br>(10738, 33974)	78.4<br>(42, 132.1)	0.5<br>(-0.9, 1.9)
Serbia	8392<br>(4470, 14097)	78.3<br>(41.8, 132.8)	8942<br>(4777, 15046)	78.4<br>(41.8, 133)	0.1<br>(-1.3, 1.5)
Slovakia	4448<br>(2388, 7507)	78.1<br>(41.5, 131.6)	5564<br>(2989, 9314)	78.3<br>(41.7, 131.7)	0.2<br>(-1.1, 1.7)
Slovenia	1798<br>(964, 3044)	78.1<br>(41.6, 132)	2194<br>(1165, 3688)	78.2<br>(41.9, 132)	0.2<br>(-1.1, 1.6)
Eastern Europe	***274886<br>(147592, 467992)***	***106.3<br>(56.9, 180.1)***	***287907<br>(154069, 486859)***	***106.4<br>(57.1, 180.3)***	***0.1<br>(-0.3, 0.5)***
Belarus	12531<br>(6688, 21171)	105.9<br>(56.3, 179)	13051<br>(6948, 21900)	106<br>(56.3, 180.7)	0.1<br>(-1.3, 1.6)
Estonia	1913<br>(1020, 3244)	105.9<br>(56.4, 179.1)	1838<br>(980, 3073)	106.2<br>(56.4, 181)	0.2<br>(-1, 1.5)
Latvia	3341<br>(1779, 5641)	105.7<br>(56.3, 180.1)	2757<br>(1461, 4615)	105.9<br>(56.4, 180.3)	0.2<br>(-0.9, 1.5)
Lithuania	4641<br>(2493, 8006)	110.2<br>(59.4, 189)	4199<br>(2256, 7132)	111.2<br>(59.7, 188.9)	0.9<br>(-3.1, 5.4)
Moldova	4861<br>(2591, 8276)	105.6<br>(56.3, 179.7)	5033<br>(2699, 8467)	106<br>(56.5, 180.1)	0.4<br>(-0.9, 1.7)
Russia	180092<br>(96683, 307472)	105.1<br>(56.2, 178.4)	196413<br>(105293, 331393)	105.4<br>(56.5, 178.5)	0.2<br>(-0.1, 0.6)
Ukraine	67508<br>(36128, 114820)	109.3<br>(58.7, 187.7)	64615<br>(34598, 110224)	109.5<br>(58.7, 186.4)	0.1<br>(-1.1, 1.4)
**High-income**	**797288<br>(426718, 1359724)**	**75.4<br>(40.6, 128.2)**	**1099930<br>(587307, 1853994)**	**79.3<br>(42.5, 135.4)**	**5.1<br>(3.3, 6.9)**
Australasia	***16621<br>(8966, 27974)***	***73.9<br>(39.8, 125.5)***	***25906<br>(13802, 43754)***	***73.5<br>(39.6, 125.6)***	***-0.6<br>(-1.8, 0.6)***
Australia	12619<br>(6811, 21184)	67.2<br>(36.2, 113.7)	20060<br>(10701, 33789)	67.3<br>(36.1, 114.5)	0.1<br>(-1.5, 1.7)
New Zealand	4002<br>(2151, 6823)	107.6<br>(57.7, 184.7)	5846<br>(3092, 9952)	107.6<br>(57.8, 184.8)	0<br>(-1.1, 1.2)
High-income Asia-Pacific	***82348<br>(44308, 138266)***	***41.4<br>(22.2, 69.8)***	***117370<br>(62514, 196325)***	***44.3<br>(23.8, 74.7)***	***6.8<br>(4.1, 10.6)***
Brunei	99<br>(53, 170)	47.5<br>(25.3, 80.1)	214<br>(113, 361)	47.4<br>(25.3, 80.2)	-0.2<br>(-2.1, 1.8)
Japan	57174<br>(30651, 95827)	37.4<br>(20.1, 62.6)	71068<br>(37997, 118433)	37.6<br>(20.2, 62.9)	0.6<br>(0.1, 1.1)
Singapore	1888<br>(999, 3180)	57<br>(30.3, 95.5)	4041<br>(2156, 6865)	58.2<br>(31.1, 98.8)	2.1<br>(-1.9, 6.6)
South Korea	23186<br>(12333, 39890)	53.9<br>(29, 91.2)	42046<br>(22663, 70797)	58.2<br>(31.5, 98.1)	8<br>(1.1, 17.5)
High-income North America	***315679<br>(169328, 541891)***	***99.1<br>(53.4, 169.1)***	***469070<br>(248724, 795961)***	***104.4<br>(55.7, 179.7)***	***5.3<br>(1.7, 9.3)***
Canada	26741<br>(14244, 45733)	85.7<br>(45.5, 146.3)	39717<br>(21181, 67773)	85.7<br>(45.6, 146.2)	0<br>(-1.6, 1.4)
Greenland	52<br>(27, 89)	92<br>(48.9, 158.7)	61<br>(32, 105)	92.4<br>(49.3, 159.8)	0.4<br>(-1.1, 1.8)
USA	288880<br>(154988, 494638)	100.6<br>(54.2, 171.6)	429285<br>(227746, 729649)	106.5<br>(56.8, 183.6)	5.9<br>(1.9, 10.2)
Southern Latin America	***43871<br>(23537, 73358)***	***91.2<br>(49, 152.6)***	***66875<br>(36114, 111515)***	***91.2<br>(49.3, 152.7)***	***0<br>(-1.1, 1)***
Argentina	29488<br>(15822, 49357)	91.3<br>(49, 153)	44071<br>(23721, 73618)	91.2<br>(49.2, 152.5)	0<br>(-1.5, 1.3)

Chile	11306<br>(6081, 19063)	91<br>(48.9, 152)	19117<br>(10205, 31759)	91.1<br>(49.1, 153)	0<br>(-1.3, 1.5)
Uruguay	3075<br>(1651, 5120)	91.2<br>(49, 152.9)	3685<br>(1970, 6149)	91.3<br>(48.9, 153.1)	0.1<br>(-1.2, 1.5)
Western Europe	***338770<br>(181930, 575509)***	***72.1<br>(38.6, 122.1)***	***420708<br>(223049, 709937)***	***72.9<br>(39.1, 123.4)***	***1.2<br>(-0.2, 3.3)***
Andorra	46<br>(25, 77)	71.4<br>(38.4, 120.2)	76<br>(40, 129)	71.3<br>(38.2, 122.1)	-0.1<br>(-1.8, 1.4)
Austria	7993<br>(4257, 13538)	83.6<br>(44.6, 143)	9891<br>(5212, 16627)	83.6<br>(44.5, 142.7)	0.1<br>(-1.4, 1.5)
Belgium	8403<br>(4514, 14327)	68.1<br>(36.3, 115.9)	10233<br>(5396, 17147)	69.3<br>(37.1, 117.1)	1.8<br>(-2.5, 6.8)
Cyprus	588<br>(312, 997)	71.3<br>(37.9, 120.8)	1146<br>(606, 1941)	71.3<br>(37.9, 120.4)	0<br>(-1.7, 1.7)
Denmark	3855<br>(2077, 6508)	60.4<br>(32.3, 102.9)	4346<br>(2275, 7317)	58.5<br>(31, 99.4)	-3.1<br>(-9.3, 1.7)
Finland	4264<br>(2261, 7186)	69.8<br>(37.1, 117.3)	5265<br>(2795, 8825)	71.4<br>(38.1, 119.3)	2.2<br>(-2.6, 7.4)
France	40412<br>(21700, 67803)	59.4<br>(31.5, 100)	50257<br>(26590, 84032)	59.5<br>(31.7, 100.1)	0.1<br>(-1.7, 1.8)
Germany	59959<br>(31870, 103397)	58.5<br>(31.3, 101.8)	71601<br>(38198, 120236)	61.8<br>(32.7, 104.8)	5.6<br>(-1.1, 16.7)
Greece	13731<br>(7264, 23657)	110.3<br>(58.3, 190)	15636<br>(8350, 27039)	110.3<br>(58.4, 188.8)	0<br>(-1.2, 1.3)
Iceland	124<br>(66, 211)	46<br>(24.7, 77.8)	191<br>(103, 321)	46.8<br>(25.2, 78.6)	1.7<br>(-2.3, 6.5)
Ireland	2638<br>(1408, 4488)	71.2<br>(38.1, 120.3)	4130<br>(2171, 6968)	71.2<br>(37.7, 120)	0<br>(-1.5, 1.4)
Israel	2946<br>(1573, 5000)	61.9<br>(33, 105.1)	5827<br>(3080, 9816)	62<br>(32.9, 104.7)	0.1<br>(-1.7, 1.8)
Italy	66937<br>(35911, 115433)	94.2<br>(50.9, 162.4)	79652<br>(42330, 138475)	94.4<br>(50.5, 163.8)	0.2<br>(-1.1, 1.6)
Luxembourg	340<br>(180, 573)	70.9<br>(37.9, 120.6)	537<br>(284, 904)	71<br>(38.1, 119.7)	0.1<br>(-1.5, 1.7)
Malta	293<br>(155, 498)	71.2<br>(38.2, 120.6)	420<br>(221, 699)	71.2<br>(38.1, 120.2)	0<br>(-1.6, 1.6)
Netherlands	11391<br>(6044, 19258)	63.9<br>(34.1, 108.5)	14358<br>(7580, 24165)	64<br>(34, 108.4)	0<br>(-1.6, 1.7)
Norway	3320<br>(1764, 5624)	64.8<br>(34.6, 109.8)	4410<br>(2330, 7448)	65.9<br>(35.3, 111.9)	1.7<br>(-1.6, 4.8)
Portugal	8180<br>(4325, 13851)	69.4<br>(37.2, 117.5)	10280<br>(5413, 17318)	69.6<br>(37.1, 117.8)	0.3<br>(-1.5, 2)
Spain	31572<br>(16837, 53011)	68.3<br>(36.2, 114.7)	42910<br>(22672, 72010)	68.4<br>(36.4, 115.6)	0.1<br>(-1.6, 1.8)
Sweden	8449<br>(4511, 14416)	78.2<br>(41.9, 133.6)	10883<br>(5746, 18325)	83.2<br>(44.5, 143.3)	6.3<br>(2.1, 11.9)
Switzerland	4878<br>(2629, 8158)	56.2<br>(30.2, 94.5)	6494<br>(3462, 10816)	56.3<br>(30.2, 95.1)	0.2<br>(-1.5, 1.9)
United Kingdom	58126<br>(30741, 97055)	82.8<br>(44, 139.2)	71730<br>(38166, 121872)	84<br>(44.8, 142.7)	1.5<br>(-1.7, 5)

**Latin America and Caribbean**	**348925 (186431, 597958)**	**110.2 (58.5, 188.8)**	**665144 (354507, 1142826)**	**109.7 (58.5, 188.3)**	**-0.4 (-1.4, 0.7)**
Andean Latin America	***31948 (17118, 54731)***	***108 (57.4, 184.2)***	***64350 (34128, 110879)***	***108.2 (57.5, 185.3)***	***0.2 (-0.7, 1.1)***
Bolivia	5083 (2711, 8758)	107.4 (57.3, 182.7)	11325 (6040, 19490)	107.8 (57.3, 183.9)	0.4 (-0.9, 1.6)
Ecuador	8436 (4498, 14469)	108.1 (57.4, 183.7)	17627 (9340, 30525)	108.2 (57.5, 186.1)	0.1 (-1.3, 1.5)
Peru	18428 (9833, 31551)	108.1 (57.7, 184.8)	35399 (18798, 60860)	108.4 (57.6, 185.5)	0.2 (-1.1, 1.5)
Caribbean	***34078 (18191, 58538)***	***108 (57.3, 183.7)***	***53415 (28412, 90791)***	***108 (57.4, 183.9)***	***0 (-0.6, 0.6)***
Antigua and Barbuda	61 (32, 105)	107.8 (57.6, 184.3)	110 (59, 188)	107.9 (57.5, 184)	0.1 (-1.2, 1.4)
The Bahamas	248 (133, 427)	108.2 (57.7, 183.7)	446 (237, 764)	108.2 (57.4, 184.1)	0 (-1.2, 1.3)
Barbados	290 (155, 497)	108.3 (57.6, 185.3)	400 (211, 679)	108.3 (57.5, 184.6)	-0.1 (-1.3, 1.2)
Belize	141 (75, 243)	108 (57.4, 184.3)	386 (205, 663)	108 (57.3, 183.7)	0.1 (-1.2, 1.3)
Bermuda	74 (39, 126)	108.3 (57.3, 184.7)	95 (50, 161)	108.4 (57.4, 184.4)	0.1 (-1.1, 1.2)
Cuba	12216 (6460, 20948)	108.2 (57.2, 185)	15680 (8307, 26766)	108.4 (57.6, 185.6)	0.2 (-1.1, 1.4)
Dominica	72 (38, 122)	108 (57.7, 183.2)	84 (45, 143)	108.1 (57.5, 183.9)	0.1 (-1.2, 1.4)
Dominican Republic	6086 (3205, 10475)	108.1 (57.7, 183.8)	11304 (6017, 19374)	108.1 (57.4, 183.7)	0 (-1.3, 1.4)
Grenada	77 (41, 131)	107.9 (57.7, 184.2)	134 (72, 229)	108.1 (57.3, 185.7)	0.2 (-1.1, 1.4)
Guyana	657 (346, 1133)	107.4 (57.3, 182.4)	775 (410, 1324)	107.5 (57, 182.9)	0.2 (-1.2, 1.5)
Haiti	5037 (2682, 8613)	107 (57, 181.3)	10920 (5820, 18762)	107.1 (56.9, 182.6)	0.2 (-1.1, 1.3)
Jamaica	2156 (1147, 3676)	108.2 (57.7, 183.2)	3218 (1713, 5457)	108 (57.6, 183.3)	-0.2 (-1.5, 1.2)
Puerto Rico	3954 (2108, 6752)	108.2 (57.7, 184.7)	5029 (2671, 8485)	108.2 (57.6, 185.7)	0 (-1.2, 1.1)
Saint Lucia	118 (62, 204)	107.9 (57.3, 185.5)	219 (116, 374)	108.1 (57.3, 184.8)	0.2 (-1, 1.4)
Saint Vincent and the Grenadines	95 (50, 162)	107.9 (57.4, 183)	136 (72, 230)	107.8 (57.4, 183.5)	0 (-1.3, 1.4)
Suriname	361 (192, 622)	108.1 (57.6, 185.4)	649 (344, 1107)	107.9 (57.5, 185)	-0.1 (-1.5, 1.1)
Trinidad and Tobago	1157 (617, 1982)	107.8 (57.3, 183.5)	1769 (948, 2990)	107.9 (57.4, 183.5)	0.1 (-1.1, 1.3)
Virgin Islands	112 (59, 192)	108.3 (57.6, 184.6)	141 (74, 237)	108.3 (57.6, 186.4)	0 (-1.3, 1.2)
Central Latin America	***138960 (73678, 237828)***	***109.9 (58.6, 187.1)***	***281397 (149704, 478874)***	***110.1 (58.6, 187.6)***	***0.1 (-0.3, 0.5)***
Colombia	29037 (15546, 50202)	108.1 (57.7, 185.2)	57472 (30481, 97945)	108.5 (57.5, 184.8)	0.4 (-1, 1.8)
Costa Rica	2706 (1430, 4682)	108.2 (57.7, 184.6)	5503 (2926, 9406)	108.4 (57.7, 184.9)	0.2 (-1.2, 1.4)

El Salvador	4297<br>(2287, 7401)	108<br>(57.2, 183.5)	6500<br>(3450, 11136)	108.1<br>(57.5, 184.7)	0.1<br>(-1.1, 1.3)
Guatemala	5891<br>(3143, 10165)	107.6<br>(57.3, 182.6)	15808<br>(8380, 27385)	108<br>(57.5, 184.7)	0.4<br>(-0.9, 1.7)
Honduras	3370<br>(1799, 5779)	108<br>(57.5, 182.5)	8629<br>(4577, 14727)	108.1<br>(57.5, 185.1)	0<br>(-1.2, 1.4)
Mexico	72505<br>(38705, 122578)	111.8<br>(59.8, 190)	142388<br>(76252, 242421)	111.8<br>(60, 190)	0<br>(-0.3, 0.3)
Nicaragua	2729<br>(1452, 4641)	107.7<br>(57.2, 183.5)	6310<br>(3388, 10851)	108.1<br>(57.9, 183.7)	0.4<br>(-1, 1.7)
Panama	2187<br>(1168, 3751)	108.2<br>(57.6, 185.3)	4319<br>(2286, 7354)	108.3<br>(57.4, 184.2)	0.1<br>(-1.2, 1.3)
Venezuela	16238<br>(8634, 28048)	108.2<br>(57.7, 184.7)	34468<br>(18295, 59121)	108.3<br>(57.5, 185.1)	0.1<br>(-1.2, 1.5)
Tropical Latin America	***143939<br>(76624, 246029)***	***111.4<br>(59.1, 189.5)***	***265982<br>(143402, 454809)***	***110<br>(59.3, 188.6)***	***-1.2<br>(-3.7, 1.4)***
Brazil	140534<br>(74807, 240409)	111.4<br>(59.1, 189.5)	258804<br>(139400, 442493)	110<br>(59.3, 188.6)	-1.2<br>(-3.8, 1.4)
Paraguay	3405<br>(1809, 5768)	110.5<br>(58.9, 185.3)	7178<br>(3820, 12169)	110.6<br>(58.8, 187.1)	0<br>(-1.2, 1.2)
**North Africa and Middle East**	**258041<br>(137603, 437059)**	**100.9<br>(54.3, 170.8)**	**583994<br>(314581, 990211)**	**101.9<br>(55.2, 172.2)**	**1<br>(0, 2.1)**
Afghanistan	7158<br>(3788, 12003)	98.3<br>(52.8, 165.1)	20927<br>(10978, 35148)	98.8<br>(52.9, 166.2)	0.5<br>(-0.9, 1.8)
Algeria	18481<br>(9787, 31219)	100.5<br>(54, 169.2)	41474<br>(22252, 70290)	100.7<br>(53.9, 170.3)	0.3<br>(-1, 1.6)
Bahrain	463<br>(247, 797)	99.8<br>(53.8, 168.4)	1741<br>(923, 2989)	99.9<br>(53.6, 169.8)	0.1<br>(-1.4, 1.6)
Egypt	43156<br>(22887, 72920)	100<br>(53.3, 168.4)	86032<br>(45919, 145476)	100.3<br>(54.3, 168.7)	0.3<br>(-1, 1.7)
Iran	42553<br>(22712, 72742)	104.3<br>(55.6, 177.5)	93767<br>(50389, 159819)	104.5<br>(55.6, 177.6)	0.2<br>(-0.3, 0.6)
Iraq	11810<br>(6295, 20039)	99.4<br>(53.1, 168.5)	35441<br>(18884, 59895)	100<br>(53.2, 169.1)	0.6<br>(-0.9, 2)
Jordan	2338<br>(1243, 3971)	91.7<br>(49.1, 154.1)	8619<br>(4622, 14632)	91.5<br>(48.5, 153.8)	-0.1<br>(-1.5, 1.3)
Kuwait	1623<br>(865, 2782)	100.2<br>(53.9, 169.8)	4942<br>(2631, 8468)	100.7<br>(54, 169.4)	0.4<br>(-0.9, 1.9)
Lebanon	3104<br>(1634, 5250)	100.4<br>(53.7, 168.5)	8088<br>(4335, 13699)	100.4<br>(53.8, 169.2)	0<br>(-1.3, 1.4)
Libya	3008<br>(1586, 5103)	100.1<br>(54.2, 168.3)	6919<br>(3718, 11724)	100.1<br>(54, 168.6)	0<br>(-1.3, 1.3)
Morocco	20168<br>(10666, 34199)	100.1<br>(53.7, 168.7)	36447<br>(19662, 61622)	100.4<br>(53.9, 168.7)	0.3<br>(-1.1, 1.8)
Palestine	1322<br>(701, 2249)	100.2<br>(53.5, 169.4)	3889<br>(2058, 6588)	100.1<br>(53.5, 168.2)	-0.1<br>(-1.4, 1.1)
Oman	1443<br>(762, 2458)	99.3<br>(53.4, 167.4)	4957<br>(2633, 8472)	99.6<br>(53.6, 168.6)	0.3<br>(-1.3, 1.8)
Qatar	438<br>(229, 752)	99.2<br>(53.1, 167.5)	3342<br>(1761, 5764)	99.5<br>(53.7, 168)	0.3<br>(-1.3, 1.9)
Saudi Arabia	13722<br>(7301, 23482)	115<br>(61.6, 196.2)	45205<br>(24145, 77520)	119.1<br>(64.5, 202.4)	3.6<br>(-1.6, 10.7)
Sudan	14130<br>(7493, 24027)	100.1<br>(53.8, 168.9)	30095<br>(15994, 51094)	100.4<br>(53.3, 170.1)	0.3<br>(-0.9, 1.5)

Syria	8458 (4482, 14393)	100.5 (53.1, 169.4)	15852 (8311, 26807)	100.5 (53.5, 170.1)	0 (-1.5, 1.5)
Tunisia	6917 (3662, 11777)	100.8 (53.9, 170.5)	12787 (6889, 21661)	100.9 (54.5, 170.5)	0.2 (-1.1, 1.4)
Turkey	47673 (25468, 80541)	98.2 (52.6, 165)	87995 (47592, 151112)	99.4 (53.6, 170.4)	1.2 (-3, 5.6)
United Arab Emirates	1742 (921, 2997)	99.4 (53.4, 167.6)	12726 (6728, 22329)	99.5 (53.8, 168.9)	0.1 (-1.5, 1.5)
Yemen	8169 (4316, 13777)	98.5 (52.9, 166.2)	22206 (11814, 37916)	99.3 (53.2, 167.3)	0.8 (-0.7, 2.2)
**South Asia**	**551852 (295845, 938652)**	**65.2 (34.9, 110.3)**	**1063039 (565190, 1785376)**	**64.2 (34.3, 107.9)**	**-1.6 (-4.6, 1.5)**
Bangladesh	50008 (26571, 84242)	68.2 (36.6, 115.1)	101250 (54322, 172459)	68.5 (36.6, 116.7)	0.4 (-1.4, 2)
Bhutan	256 (135, 435)	65 (34.9, 110.3)	588 (311, 1014)	65.4 (34.9, 110.9)	0.6 (-1, 2.1)
India	443591 (237053, 753701)	64.9 (34.7, 109.8)	833930 (444148, 1404296)	63.5 (33.9, 106.7)	-2.2 (-5.8, 1.9)
Nepal	8925 (4726, 15176)	64.6 (34.5, 108.9)	17261 (9219, 29226)	65.1 (34.8, 110.5)	0.8 (-1, 2.5)
Pakistan	49072 (26375, 83273)	65 (34.8, 110.1)	110009 (58096, 187158)	65.1 (34.7, 110.8)	0.2 (-1.5, 1.9)
**Southeast Asia, East Asia, and Oceania**	**925456 (494310, 1576799)**	**58.2 (31.0, 98.7)**	**1493828 (796026, 2518595)**	**58.5 (31.2, 99.4)**	**0.5 (0.1, 0.9)**
East Asia	***673484 (359628, 1145787)***	***55.9 (29.8, 94.4)***	***1039270 (550346, 1744339)***	***55.8 (29.8, 94.3)***	***-0.1 (-0.6, 0.4)***
China	635827 (339112, 1081624)	55.6 (29.6, 93.8)	981979 (519771, 1649456)	55.5 (29.6, 93.5)	-0.2 (-0.7, 0.2)
North Korea	12139 (6352, 20493)	60.6 (31.6, 101.9)	18356 (9820, 30979)	60.6 (32.4, 102.9)	-0.1 (-1.7, 1.7)
Taiwan (Province of China)	14297 (7599, 24435)	69.5 (36.9, 118.5)	22193 (11766, 37677)	73.2 (39.1, 125)	5.3 (-0.2, 12.1)
Oceania	***3097 (1626, 5318)***	***63.7 (34, 108.4)***	***6634 (3498, 11367)***	***63.6 (34, 107.6)***	***-0.2 (-1.4, 1.1)***
American Samoa	24 (13, 42)	63.9 (34.2, 109.5)	32 (17, 55)	63.7 (34.2, 108.6)	-0.3 (-2, 1.4)
Federated States of Micronesia	46 (24, 80)	63.9 (33.9, 108.5)	59 (31, 100)	63.7 (34.1, 107.5)	-0.3 (-1.9, 1.3)
Fiji	395 (210, 677)	63.8 (34, 108.5)	565 (302, 968)	63.7 (34.2, 108.8)	-0.2 (-2, 1.5)
Guam	81 (43, 140)	64.4 (34.1, 110.1)	112 (59, 190)	64.1 (34.3, 110)	-0.4 (-2.3, 1.4)
Kiribati	37 (19, 64)	63.7 (34, 108.1)	63 (34, 107)	63.4 (33.8, 107.5)	-0.5 (-2.3, 1.3)
Marshall Islands	19 (10, 32)	64 (33.8, 109.5)	32 (17, 54)	63.8 (34.2, 108.3)	-0.3 (-1.9, 1.4)
Northern Mariana Islands	28 (15, 48)	64.4 (34.3, 109.5)	33 (17, 56)	64.1 (34.2, 109.6)	-0.5 (-2.2, 1.3)
Papua New Guinea	1925 (1010, 3301)	63.6 (34.1, 108)	4744 (2488, 8115)	63.5 (33.9, 107.4)	-0.1 (-1.8, 1.6)

Samoa	78<br>(41, 133)	64.2<br>(34.4, 109.4)	105<br>(56, 179)	64<br>(34.2, 109.4)	-0.3<br>(-1.9, 1.3)
Solomon Islands	145<br>(75, 249)	64<br>(34.2, 109)	319<br>(167, 546)	63.7<br>(34, 108.6)	-0.4<br>(-2, 1.3)
Tonga	46<br>(24, 79)	64<br>(34, 109.1)	58<br>(31, 99)	63.9<br>(34.1, 108.5)	-0.2<br>(-1.9, 1.5)
Vanuatu	69<br>(36, 118)	63.8<br>(34, 108.6)	148<br>(78, 253)	63.7<br>(33.6, 108.2)	-0.2<br>(-2, 1.8)
Southeast Asia	***248875<br>(132143, 428969)***	***65.4<br>(35, 111.2)***	***447923<br>(239081, 764424)***	***65.6<br>(35.1, 112)***	***0.3<br>(-0.2, 0.7)***
Cambodia	4541<br>(2391, 7828)	63.5<br>(34.1, 107.9)	9569<br>(5081, 16369)	64<br>(34.2, 108.6)	0.7<br>(-1, 2.3)
Indonesia	101376<br>(54456, 173272)	67.3<br>(36.5, 114.1)	177511<br>(95775, 301554)	67.5<br>(36.4, 114.6)	0.3<br>(-0.3, 0.8)
Laos	1909<br>(1005, 3265)	64<br>(34.2, 109.4)	3987<br>(2093, 6844)	64.3<br>(34.3, 109.6)	0.5<br>(-1.2, 2.2)
Malaysia	9273<br>(4882, 15967)	64.4<br>(34.3, 109.7)	20250<br>(10709, 34818)	64.5<br>(34.4, 110.7)	0.2<br>(-1.6, 1.9)
Maldives	93<br>(49, 159)	64<br>(34, 109.1)	318<br>(171, 551)	64.6<br>(34.7, 110.3)	0.9<br>(-0.8, 2.7)
Mauritius	660<br>(352, 1131)	64<br>(34.4, 108.7)	977<br>(525, 1650)	63.9<br>(34.3, 108.9)	-0.1<br>(-1.6, 1.5)
Myanmar	21046<br>(11075, 36167)	63.7<br>(34, 108.1)	33959<br>(17989, 58173)	64.1<br>(34.1, 109.6)	0.6<br>(-1.1, 2.1)
Philippines	30819<br>(16054, 53378)	64<br>(34, 109.1)	60539<br>(32313, 104251)	64.1<br>(34, 109.2)	0.2<br>(-1.5, 2)
Sri Lanka	9873<br>(5202, 16882)	64.2<br>(34.3, 109.3)	15229<br>(8119, 25871)	64.4<br>(34.2, 109.9)	0.2<br>(-1.4, 1.7)
Seychelles	42<br>(22, 72)	64.4<br>(34.6, 109.6)	73<br>(39, 124)	64.5<br>(34.5, 109.6)	0.1<br>(-1.5, 1.8)
Thailand	33874<br>(17804, 58383)	64.4<br>(34.1, 110.3)	56870<br>(29988, 96832)	64.6<br>(34.4, 110.8)	0.3<br>(-1.5, 2)
East Timor	362<br>(191, 613)	63.5<br>(33.6, 107.8)	645<br>(340, 1104)	63.9<br>(34, 108.8)	0.5<br>(-1.2, 2.3)
Vietnam	34677<br>(18083, 60009)	64.2<br>(34.1, 109.8)	67408<br>(36106, 115138)	64.5<br>(34.6, 110.1)	0.5<br>(-1, 2.1)
**Sub-Saharan Africa**	**280041<br>(149448, 478162)**	**84.6<br>(45.5, 142.8)**	**615748<br>(328576, 1052288)**	**85.0<br>(45.7, 143.8)**	**0.4<br>(0.1, 0.8)**
Central sub-Saharan Africa	***30526<br>(16253, 52390)***	***83.1<br>(44.9, 141.4)***	***71326<br>(38135, 121636)***	***83.6<br>(45.2, 141.9)***	***0.6<br>(-0.5, 1.7)***
Angola	5712<br>(3037, 9807)	83.4<br>(45.1, 141.3)	15560<br>(8363, 26802)	83.7<br>(45.3, 142.2)	0.4<br>(-0.9, 1.7)
Central African Republic	1566<br>(834, 2691)	83.1<br>(44.6, 141)	2915<br>(1549, 4996)	83.6<br>(45, 141.7)	0.6<br>(-0.8, 2)
Congo (Brazzaville)	1374<br>(732, 2371)	83.2<br>(44.9, 141.3)	3356<br>(1810, 5738)	83.8<br>(45.5, 142.4)	0.7<br>(-0.7, 2.1)
DR Congo	21027<br>(11200, 36141)	82.9<br>(44.8, 141.1)	47488<br>(25346, 81094)	83.5<br>(45.1, 142)	0.7<br>(-0.8, 2.2)
Equatorial Guinea	235<br>(126, 404)	83.1<br>(45, 141.5)	776<br>(420, 1342)	83.9<br>(45.3, 143.1)	0.9<br>(-0.6, 2.4)
Gabon	613<br>(332, 1056)	83.6<br>(45.3, 141.8)	1231<br>(663, 2130)	83.9<br>(45.2, 143.1)	0.4<br>(-1.3, 2)
Eastern sub-Saharan Africa	***104238<br>(55685, 177104)***	***85.3<br>(45.9, 143.7)***	***230409<br>(122933, 392860)***	***85.8<br>(46, 144.6)***	***0.7<br>(0.2, 1.1)***

Burundi	3030 (1610, 5246)	84.3 (45.6, 144)	6207 (3318, 10670)	84.4 (45.5, 143.6)	0.1 (-1.4, 1.5)
Comoros	258 (136, 441)	84 (45.1, 143.2)	514 (276, 881)	84.5 (45.6, 143.9)	0.7 (-0.8, 2.1)
Djibouti	278 (148, 478)	84.6 (45.5, 143.4)	820 (439, 1423)	84.8 (45.6, 145.3)	0.2 (-1.3, 1.7)
Eritrea	1498 (799, 2579)	82.8 (44.7, 140.5)	3600 (1919, 6146)	83.8 (45.2, 142)	1.2 (-0.5, 2.8)
Ethiopia	28234 (14950, 47334)	87.3 (46.8, 145.9)	60875 (32185, 103529)	88.3 (47.3, 148.7)	1.2 (0.3, 2)
Kenya	12446 (6567, 20962)	88.3 (47.2, 148.5)	31524 (16732, 53529)	88.3 (47.1, 148.2)	0 (-0.3, 0.3)
Madagascar	6692 (3601, 11498)	84.2 (45.4, 143.1)	15353 (8212, 26308)	84.4 (45.3, 144)	0.3 (-1.2, 1.8)
Malawi	5379 (2886, 9241)	83.9 (45.2, 142.9)	10081 (5391, 17445)	84.3 (45.4, 144)	0.5 (-1.1, 1.9)
Mozambique	8055 (4304, 13832)	83.7 (45.2, 142.2)	16383 (8832, 28232)	83.9 (45.6, 143.2)	0.1 (-1.3, 1.5)
Rwanda	3969 (2102, 6870)	84.2 (45.2, 143.2)	7853 (4214, 13532)	84.4 (45.5, 143.3)	0.2 (-1.2, 1.8)
Somalia	3839 (2039, 6567)	83.9 (45.2, 142.1)	9304 (4936, 16085)	84.2 (45.4, 143.7)	0.3 (-1.2, 1.8)
South Sudan	3309 (1773, 5664)	83 (45, 140.1)	5389 (2887, 9255)	83.5 (45, 141.8)	0.6 (-1.1, 2)
Tanzania	14123 (7516, 24305)	83.9 (45.3, 142)	31817 (16868, 54823)	84.5 (45.5, 143.3)	0.6 (-0.8, 2)
Uganda	8902 (4762, 15205)	83.2 (45.2, 140.7)	20577 (10977, 35161)	84.2 (45.5, 142.7)	1.2 (-0.4, 2.8)
Zambia	4171 (2234, 7166)	84.3 (45.5, 142.4)	9968 (5270, 17122)	84.3 (45.3, 143)	0.1 (-1.4, 1.6)
Southern sub-Saharan Africa	***34913 (18655, 59023)***	***87 (46.6, 145.9)***	***62571 (33349, 105947)***	***86.9 (46.5, 145.7)***	***-0.1 (-0.5, 0.4)***
Botswana	751 (404, 1283)	83.7 (45.1, 142)	1757 (946, 3027)	83.6 (45.1, 141.4)	-0.2 (-1.7, 1.2)
Lesotho	1106 (590, 1895)	83.7 (45.1, 142.4)	1397 (748, 2417)	83.1 (45, 141.7)	-0.7 (-2.1, 0.6)
Namibia	849 (453, 1464)	83.8 (45.1, 142.7)	1657 (889, 2842)	83.8 (45.1, 142.6)	0 (-1.5, 1.5)
South Africa	26185 (13918, 44193)	88 (46.9, 147.6)	47848 (25490, 80580)	87.8 (46.8, 146.9)	-0.2 (-0.7, 0.3)
Swaziland	424 (226, 731)	83.8 (45, 142.4)	746 (400, 1282)	83.1 (44.8, 142.1)	-0.8 (-2.3, 1)
Zimbabwe	5597 (2983, 9573)	83.9 (45.4, 143.1)	9165 (4870, 15862)	83.7 (45.1, 141.9)	-0.2 (-1.8, 1.2)
Western sub-Saharan Africa	***110364 (58945, 189595)***	***83.7 (45.1, 141.4)***	***251442 (134319, 431232)***	***84.1 (45.1, 143)***	***0.5 (-0.2, 1.2)***
Benin	2526 (1349, 4353)	83.2 (44.9, 141.5)	6615 (3556, 11402)	84 (45.4, 142.7)	1 (-0.6, 2.6)
Burkina Faso	5046 (2691, 8668)	83.1 (44.7, 141.8)	11968 (6420, 20475)	84.3 (45.4, 143.6)	1.5 (-0.1, 3.3)
Cameroon	5762 (3095, 9943)	83.3 (45, 141.5)	16497 (8841, 28269)	84.1 (45.2, 142.8)	0.9 (-0.6, 2.6)
Cape Verde	209 (112, 360)	84.5 (45.5, 143.9)	446 (239, 771)	84.8 (45.7, 144.1)	0.3 (-1, 1.7)
Chad	3253 (1745, 5617)	83.4 (44.8, 141.1)	7554 (4031, 12925)	83.7 (45.1, 141.7)	0.4 (-1.1, 1.9)

Côte d'Ivoire	6623<br>(3534, 11360)	83.3<br>(45, 141.3)	15535<br>(8307, 26852)	84.1<br>(45.5, 142.4)	0.9<br>(-0.5, 2.4)
The Gambia	531<br>(284, 909)	83.6<br>(45.3, 141.2)	1274<br>(683, 2201)	83.9<br>(45.4, 142.8)	0.3<br>(-1.2, 1.9)
Ghana	8657<br>(4644, 14916)	84<br>(45.2, 143.1)	20516<br>(11018, 35239)	84.4<br>(45.4, 143.3)	0.4<br>(-1.1, 1.9)
Guinea	3612<br>(1931, 6191)	83.7<br>(45.3, 142.7)	6833<br>(3653, 11805)	84.1<br>(45.3, 142.7)	0.5<br>(-1, 2.1)
Guinea-Bissau	535<br>(287, 926)	83.5<br>(45.1, 142.2)	1083<br>(579, 1870)	83.9<br>(45.4, 142.7)	0.5<br>(-0.9, 1.8)
Liberia	1151<br>(619, 1976)	83.2<br>(45, 141)	2864<br>(1541, 4894)	83.2<br>(44.9, 141.3)	-0.1<br>(-1.5, 1.4)
Mali	4800<br>(2589, 8231)	83.1<br>(45, 141.5)	10874<br>(5806, 18710)	83.9<br>(44.9, 142.1)	0.9<br>(-0.8, 2.4)
Mauritania	1199<br>(640, 2060)	83.8<br>(45.3, 142.5)	2410<br>(1296, 4131)	84.3<br>(45.2, 143.6)	0.5<br>(-1.1, 2.1)
Niger	4100<br>(2181, 7038)	84<br>(45.5, 143.7)	10456<br>(5560, 17928)	84.3<br>(45.3, 144)	0.3<br>(-1.4, 2)
Nigeria	54003<br>(28841, 92462)	83.8<br>(45.1, 140.9)	117772<br>(62717, 202295)	84<br>(45.1, 143.1)	0.2<br>(-1.1, 1.8)
São Tomé and Príncipe	68<br>(36, 118)	84.4<br>(45.4, 143.4)	133<br>(71, 228)	84.5<br>(45.6, 143.2)	0.2<br>(-1.4, 1.7)
Senegal	4112<br>(2207, 7032)	83.7<br>(45, 141.8)	9036<br>(4850, 15518)	84.1<br>(45.1, 143.2)	0.5<br>(-0.9, 1.9)
Sierra Leone	2268<br>(1222, 3901)	83.7<br>(45.3, 142.3)	4770<br>(2539, 8161)	83.9<br>(45.2, 142.2)	0.3<br>(-1.3, 1.8)
Togo	1905<br>(1018, 3245)	83.7<br>(45, 141.4)	4804<br>(2557, 8238)	84.3<br>(45.2, 143.3)	0.7<br>(-1, 2.4)
